# Optically Responsive Field-Effect Transistors Based on Graphene/Diarylethene Hybrid Films

**DOI:** 10.1021/acsomega.6c07553

**Published:** 2026-08-05

**Authors:** Francesco Vinti, Adrián Tamayo, Yeonsu Jeong, Piotr Cieciórski, Wojciech Danowski, Paolo Samorì, Loredana Latterini

**Affiliations:** † Nano4Light Lab, Department of Chemistry, Biology and Biotechnology, University of Perugia, via Elce di Sotto 8, Perugia 06123, Italy; ‡ University of Strasbourg & CNRS, ISIS & icFRC, 8 allee Gaspard Monge, 67000 Strasbourg, France; § Institute of Quantum Science, Department of Physics, 26718 Inha University, 100 Inha-ro, Michuhol-gu, Incheon 22212, Republic of Korea; ∥ Faculty of Chemistry, University of Warsaw, Ludwika Pasteura 1, 02-093 Warsaw, Poland

## Abstract

Photochromic molecules are emerging as key components in advanced optoelectronic systems owing to their ability to undergo reversible light-induced structural transformations that modulate their optical and electronic properties. Here, we investigate the optical response and photoswitching behavior of two prototypical dioctadecyl-substituted diarylethenes (DTEs) in solution and as ultrathin films physisorbed onto graphene crystals. Photoswitching efficiency was quantified spectrophotometrically in both environments, revealing efficient and reversible interconversion between the open and closed isomeric forms. When assembled into physisorbed monolayers on graphene, these photoresponsive molecules provide a remote means of tuning the electronic properties of the hybrid interface. The morphology, optical characteristics, and electronic structure of DTE/graphene assemblies were systematically characterized and correlated with variations in drain current measured in field-effect transistors (FETs) incorporating the hybrid films as active channel components.

## Graphical abstract

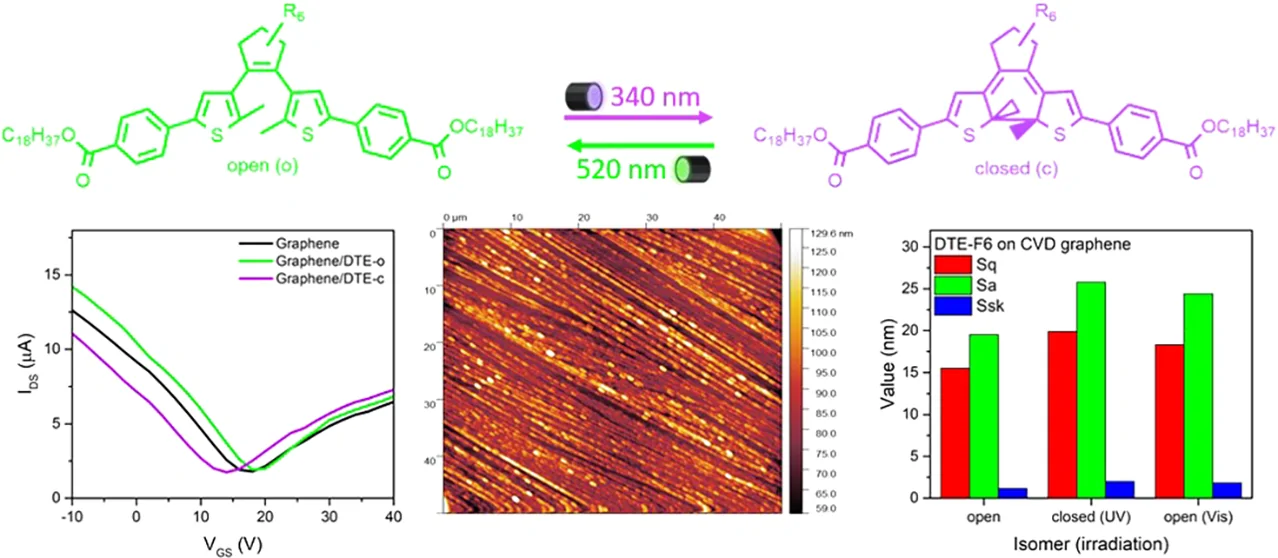

## 1 Introduction

Graphene and related materials possess outstanding physical and chemical properties that are, unfortunately, hardly tunable. Their interfacing with molecular materials represents a versatile tool for boosting their functional complexity and generating hybrid structures. In particular, upon decorating the surfaces of graphene with molecular switches, materials capable of responding to multiple independent stimuli can be tailored.

Among the different external inputs used to modulate a material’s properties, light has emerged due to its spatiotemporal resolution, wavelength selectivity, and the possibility of remote control without physical contact. Over the years, different ways to achieve and exploit a material’s photoactivation^1–3^ have been studied and developed, starting from the photovoltaic devices,^4^ molecular machines and motors,^5^ molecular photoswitches,^6,7^ photoresponsive electronic devices,^8–10^ and many more.^11,12^

Photochromic molecules are photoresponsive organic compounds that are capable of undergoing efficient and reversible isomerization between different states when exposed to light at specific wavelengths. While the greatest majority of photochromic molecules, such as azobenzenes and spiropyrans, possess only one thermally stable state, diarylethene (DTE)’s open and closed isomers are both thermodynamically stable. Such a special characteristic is extremely important for digital applications in the context of realizing nonvolatile memories.

Field-effect transistors (FETs) are the key elements of today’s electronics. In analogy to most electronic devices, FETs are monofunctional as they respond to one stimulus, i.e., the voltage that is applied to the gate electrode. The fabrication of devices responding independently to multiple stimuli has been the subject of intensive research over the past decade because of its relevance both for fundamental studies and for advanced applications. The latter holds paramount importance for emerging computing and sensing paradigms, including neuromorphic systems,^13^ where the ability to process and integrate information from multiple independent inputs can enhance device functionality and computational versatility.

In this work, we fabricated organic–inorganic hybrid films upon physisorption of a DTE molecular film onto the basal plane of graphene. The multiscale properties of this hybrid have been investigated at multiple length scales, making use of several techniques. UV–vis spectrophotometry provided molecular-scale insight into the ability of the DTEs to undergo efficient and reversible photoisomerization; atomic force microscopy (AFM) offered direct information on the morphology of the DTE films physisorbed onto graphene; whereas the light-responsiveness of the hybrid material has been investigated through its integration as a channel component in a FET, whose electrical behavior was assessed when exposed to light at different wavelengths.

## 2 Materials and Methods

### 2.1 Film Preparation

Thin films of the two DTEs were obtained by spin coating at 1000 rpm for 30 s, using solutions of the two DTEs in cyclohexane; the depositions were performed over a quartz slide, opportunely pretreated. The pretreatment consisted of two washing steps in ethanol and then acetone, under ultrasonication, followed by 30 min in an ozone cleaning chamber, and concluded by an overnight soak in cyclohexane, to improve the wettability of the substrate.

### 2.2 Device Fabrication

The chemical vapor deposition (CVD) graphene was wet-transferred via a polymer-assisted transfer from the Cu foil onto SiO_2_ (90 nm)/Si substrates using a poly­(methyl methacrylate) (PMMA, Sigma-Aldrich) stamp. The Cu was subsequently removed by sacrificial etching by using a commercial copper etchant (Sigma-Aldrich).

After transferring the graphene onto SiO_2_ (90 nm)/Si substrates, source/drain (S/D) electrodes of Cr/Au (2/50 nm) were patterned by photolithography, followed by thermal evaporation and a lift-off process in warm acetone (40 °C). The channel length (*L*) and width (*W*) of the transistors were 40 and 50 μm, respectively.

The CVD graphene devices were annealed overnight under ultrahigh vacuum at 80 °C. All devices were stored and measured in a nitrogen-filled glovebox using a probe station.

After this procedure, the standard electrical characteristics of pristine graphene were measured. The transfer characteristics of the graphene device are shown in Figure 6a (black line). They reveal a charge neutrality point (*V*_CNP_) near 20 V, indicating slight p-type doping, likely induced by the transfer process and possible residues on the graphene surface. The reproducibility of the results was verified by using more than five different graphene devices, showing excellent qualitative and quantitative agreement.

The DTE/graphene hybrids were prepared by depositing the DTE layer upon spin coating a 45 μL drop of a 2 mM solution in cyclohexane onto a CVD-graphene transistor, followed by thermal annealing (20 min at 60 °C).

In the final device, graphene plays a dual role; first, it acts as the channel material providing the conductive pathway between the source and the drain electrodes of the FET; second, it serves as the reversibly dopable material whose electronic properties can be modulated upon interaction with the two DTE’s isomers.

### 2.3 UV–Vis Characterization

For solution analysis, qualitative and quantitative analyses were used with two different instruments, both with a double beam and a monochromator for the selection of wavelength. They are a CARY 1 UV–vis from Varian, and a V-630 UV–vis–NIR spectrometer from Jasco. Samples were prepared in dilute solutions and analyzed in a standard 1 cm quartz cuvette.

For film analysis and kinetic measurements, a CARY 8454 UV–vis from Agilent was used, which is a single beam instrument coupled with a charge-coupled device (CCD) detector, equipped with a vertical sample holder for films.

### 2.4 Raman Characterization

Raman measurements were performed using an Olympus IX73 instrument assembled by Crisel, equipped with a S&I MonoVista CRS7 spectrometer, and an inVia instrument by Renishaw, both studied for solid samples. The lasers used are the 532 nm and the 785 nm. The samples, powder, and film were deposited on a quartz slide and directly irradiated with the lasers.

### 2.5 AFM Analysis

The AFM instrument used for this work is a Dimension ICON AFM from Bruker, working in tapping mode with a TESPA-V2 tip.

Thin films were prepared on top of CVD graphene on silicon oxide/silicon wafers or mica.

### 2.6 Device Characterization

The bottom-gate, top-contact devices were electrically characterized using a Keithley 2636 under an N_2_ atmosphere. For light-induced photoswitching, a polychrome V monochromator was used to irradiate the FETs from the top. UV irradiation (340 nm, 0.08 mW·cm^–2^) was applied for 20 min, followed by visible-light irradiation (520 nm, 0.56 mW·cm^–2^) for 120 min. During this characterization, the UV laser power was lowered to avoid the formation of the photoirreversible annulated byproduct. Its formation would have reduced the device efficiency, and since we did not perform any UV–vis spectroscopy during these measurements, it would have been very difficult to detect it.

## 3 Results and Discussion

Two DTE derivatives equipped with octadecyl side chains were designed and synthesized. Such linear alkyl chains have been chosen in view of their propensity to physisorb onto the basal plane of graphene, forming crystalline lamellar structures, enabling the positioning of the DTEs onto the two-dimensional (2D) materials with atomic precision, thereby forming a superlattice. The DTE core is well-known for undergoing photoisomerization between two different isomers, i.e., a cross-conjugated open form, which is typically colorless, and the cyclized fully conjugated isomer that usually absorbs in the visible region. Upon irradiation with UV or visible light, the molecule undergoes efficient isomerization between the two forms with remarkably high photoconversion to the respective isomer. Also, both isomers have high thermodynamic stability, as evidenced by a negligible thermal back-reaction.^14–16^

The detailed synthetic procedures for the chosen DTE derivatives and their characterizations are described in the Supporting Information (Figures S1–S10). Figure 1 shows the chemical structures of the two DTEs employed in this work. The key structural difference between the two molecules is the presence of six fluorine atoms in the main core of the DTE structure (DTE-F6), compared to the nonfluorinated analogue bearing hydrogen substituents (DTE-H6). The former was selected since the use of a perfluorocyclopentane core in DTEs is known to offer a more efficient and reversible ring-closing/ring-opening photochromism.^17^

**Figure 1:**
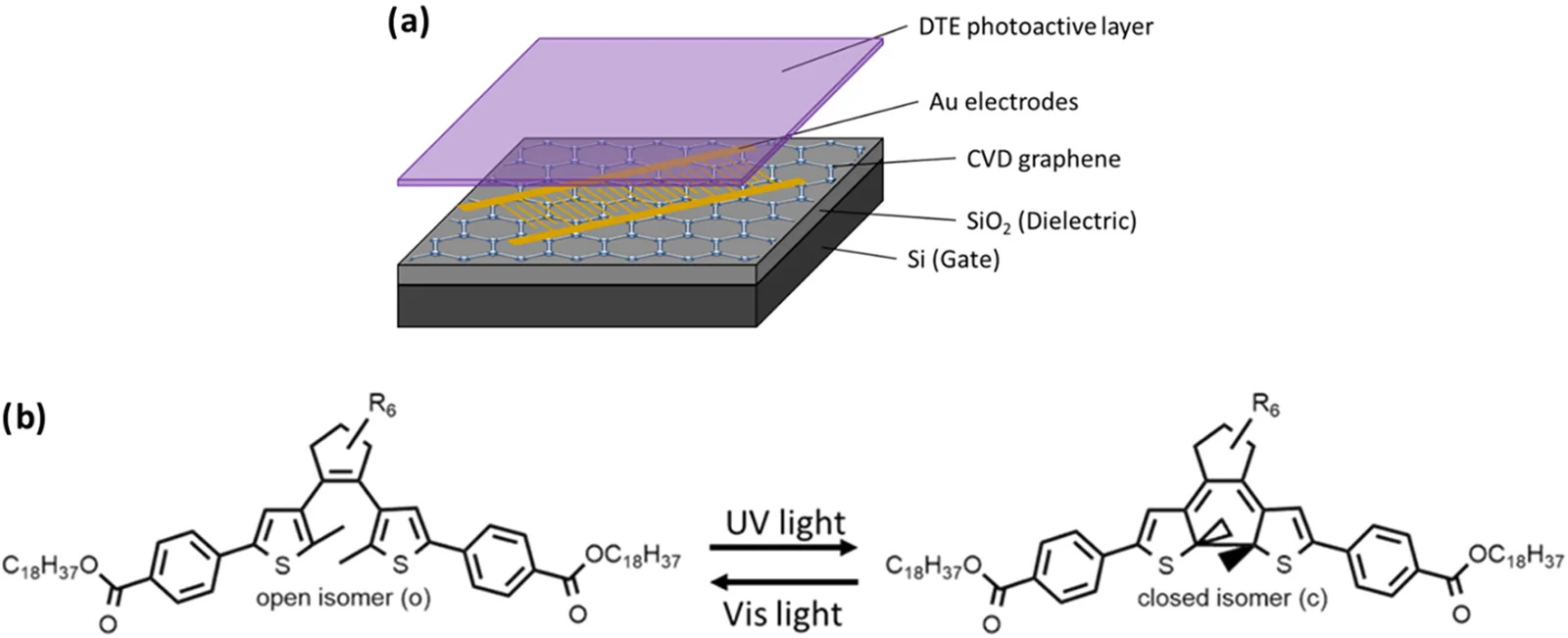
Structure of (a) a graphene/DTE FET; (b) the two DTEs used in this work, R = H (DTE-H6), R = F (DTE-F6), and their characteristic isomerization.

The absorbance spectra of the open and closed isomers shown in Figure S11 indicate that fluorination induces a red shift and a more pronounced visible absorption band in the closed isomer, resulting in enhanced spectral contrast between the open and closed states. The quantum yields of photoisomerization as well as photoconversions to the respective isomer at each photostationary state (PSS) are presented in Table 1. The combined data indicate that the photochemical behavior of both compounds is comparable to related structures,^18,19^ and that the long alkyl side chains have only a marginal impact on the light-responsiveness of both molecules in solution.

**Table 1:** Isomerization Quantum Yields and Percentage Conversion Values, after the First Isomerization Cycle, of Coloration (Col) and Decoloration (Decol) Photoreactions of Various Samples of DTEs

sample	Φ_Col_	% closed_PSS_	Φ_Decol_	% open_recovery_
DTE-H6 solution	0.75	95	0.0012	96
DTE-F6 solution	0.43	94	0.0014	99
DTE-H6 on quartz	0.051	27	0.0013	85
DTE-F6 on quartz	0.13	60	0.0015	98
DTE-H6 on CVD	0.075	35	0.0011	97
DTE-F6 on CVD	0.20	55	0.0012	99

Graphene was chosen as the electroactive platform owing to its outstanding in-plane conductivity, high charge carrier mobility, and its ability to support ordered molecular adsorption and surface functionalization, making it an attractive platform for integrating photoresponsive molecular systems.^8,20^

The DTE/graphene hybrids were prepared by depositing the DTE layer by spin coating a 45 μL drop of a 2 mM solution in cyclohexane onto CVD-grown graphene supported on transparent quartz substrates, which was chosen in order to enable optical characterization under UV–vis irradiation. UV irradiation (300 and 340 nm) triggers the isomerization from the ring-open form (transparent samples) to the ring-closed form, resulting in a color change of the samples. Upon reaching the PSS, the two films were irradiated with visible light (520 nm), in order to regenerate the ring-open isomer. For both photoswitches in solution, prolonged irradiation with green light led to full recovery of the initial absorption spectra, consistent with a near quantitative degree of photoconversion upon ring-opening isomerization. In Figures 2 and S12, the absorption spectra of the two DTE/graphene hybrids recorded after successive irradiation steps are collected. Notably, the fluorinated derivative (DTE-F6) exhibits efficient photoswitching behavior even under significantly lower irradiation intensity compared to DTE-H6, indicating enhanced photoswitching efficiency upon fluorination.

**Figure 2:**
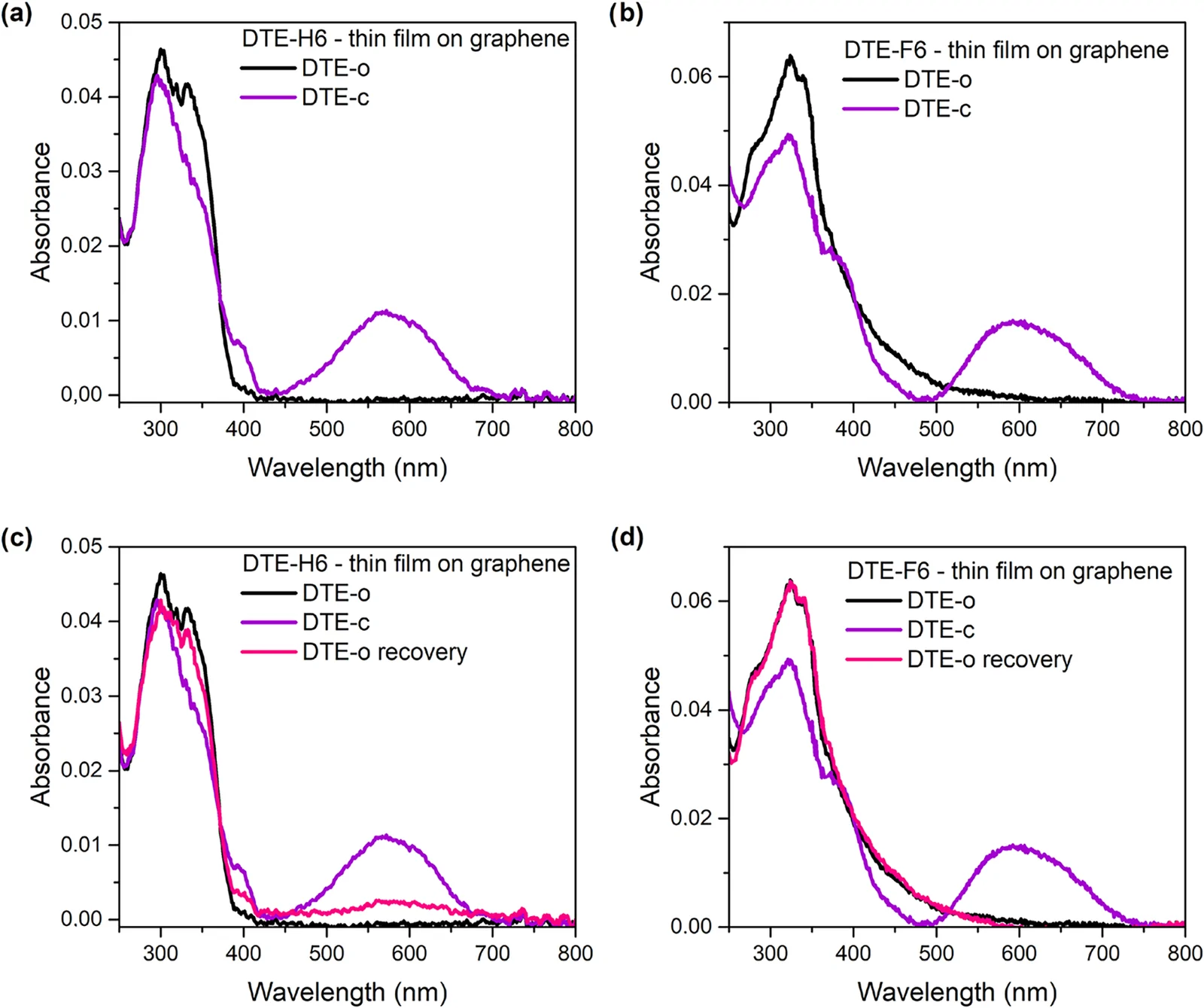
Optical behavior of thin films on CVD graphene. Absorption spectra of: (a) DTE-H6 film under UV irradiation (50 s), 300 nm, 0.39 mW·cm^–2^; (b) DTE-F6 under UV irradiation (5 min), 340 nm, 40 μW·cm^–2^; (c) DTE-H6 under vis irradiation (240 min), 520 nm, 9.86 mW·cm^–2^; and (d) DTE-F6 under vis irradiation (60 min), 520 nm, 0.52 mW·cm^–2^. The choice of UV irradiation wavelength was dictated by the distinct absorption spectra of the two molecules.

The major difference between the two photoswitches regards the photoisomerization quantum yield of the photoconversion. In fact, the fluorinated compound showed a much higher yield than its hydrogenated counterpart. This is likely due to the lower affinity of perfluoroalkanes toward the basal plane of graphene than hydrocarbons,^21^ which ensures a higher isomerization efficiency.

Figure 3 shows that the fluorinated DTE derivative is characterized by a better conversion for both processes, as evidenced by a conversion of 50–60% to the closed isomer under UV light and almost quantitative recovery of the open isomer under visible-light irradiation. In stark contrast, the hydrogenated derivative shows significantly lower photoconversion to the respective isomers at each PSS and a more pronounced photofatigue amounting to ∼5% degradation over three isomerization cycles.

**Figure 3:**
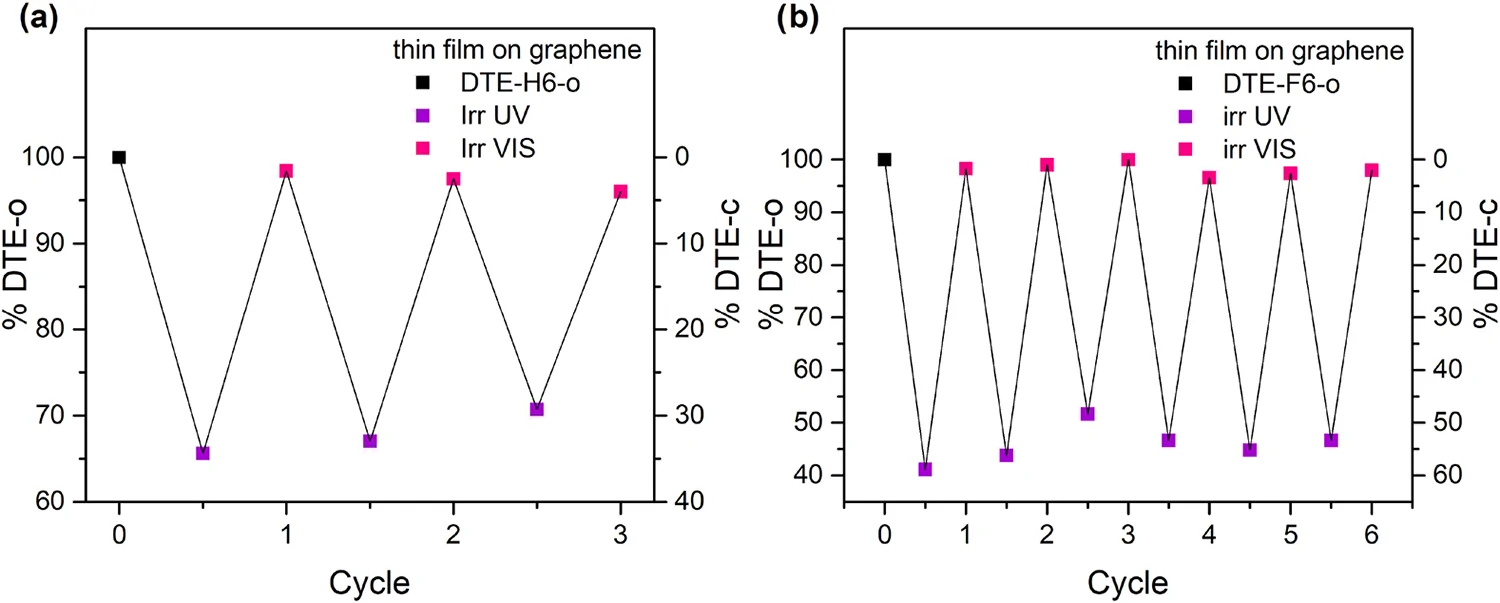
Percentage values of the conversion during few subsequent cycles; (a) DTE-H6; (b) DTE-F6.

Taken together, these data demonstrate that DTE-F6 exhibits superior photochemical properties relative to its perhydro counterpart, especially in the solid state as thin films deposited on graphene. As observed in previous reports,^22,23^ when a molecular system is deposited as a thin film, its absorption and electronic properties depend on its crystallinity. To cast light onto this issue, the morphology of the two DTE films physisorbed onto CVD graphene, before and after a full isomerization cycle, was explored by AFM imaging.

Figure 4a–f shows that the change in the morphology occurring upon open-closed isomerization is noticeable for DTE-H6, in line with previous works.^24^ Conversely, DTE-F6 displays a more limited morphological change, as shown in Figure 4g–l. AFM data, measuring the height of a groove traced on the film surface, reveal that DTE-H6 and DTE-F6 films have a thickness corresponding to 20 and 100 nm, respectively. This difference is expected due to the presence of the fluorine atoms that hinder stacked and flat deposition. Upon irradiation, DTE-H6 samples are characterized by a major morphological change, while DTE-F6 films show an imperceptible morphological modification. This is visualized by the height parameters, obtained from the statistical analysis of the AFM images, portrayed in Figure 5, which better explain the morphological modification observed in the topography. *S*_q_ is the standard deviation of the height distribution, *S*_a_ is the arithmetic mean of the height distribution, and *S*_sk_ indicates the skewness of the surface. Taken together, these data indicate that upon isomerization, the DTE-F6 films do not undergo a significant structural change like the DTE-H6 ones. This morphology change is also correlated with the time difference observed for the photorecovery, shown in Figure 2.

**Figure 4:**
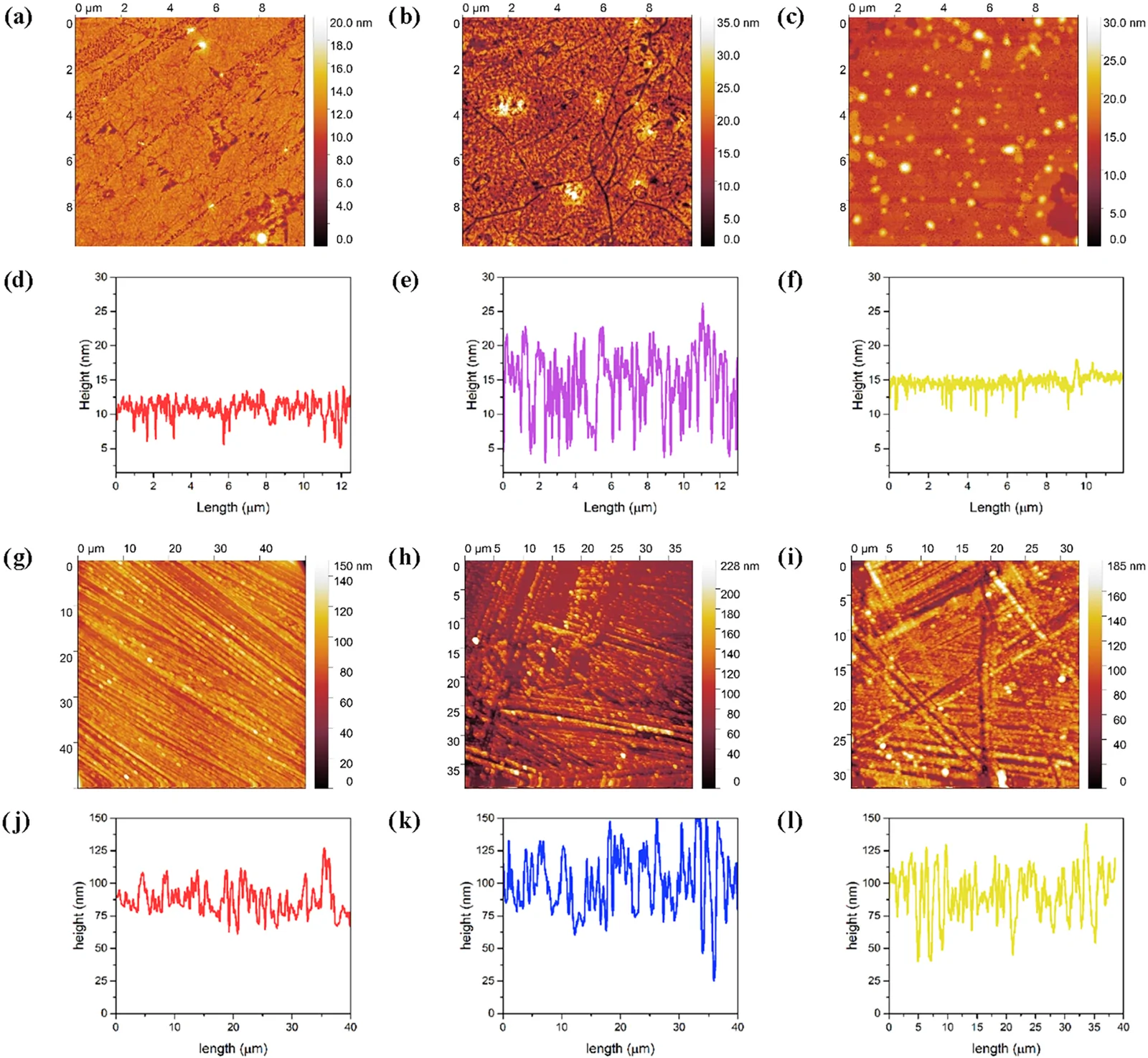
Topographical AFM images and topography profiles of (a–f) DTE-H6; (a, d) open isomer; (b, e) closed isomer; (c, f) open recovery; (g–l) DTE-F6; (g, j) open isomer; (h, k) closed isomer; and (i, l) open recovery.

**Figure 5:**
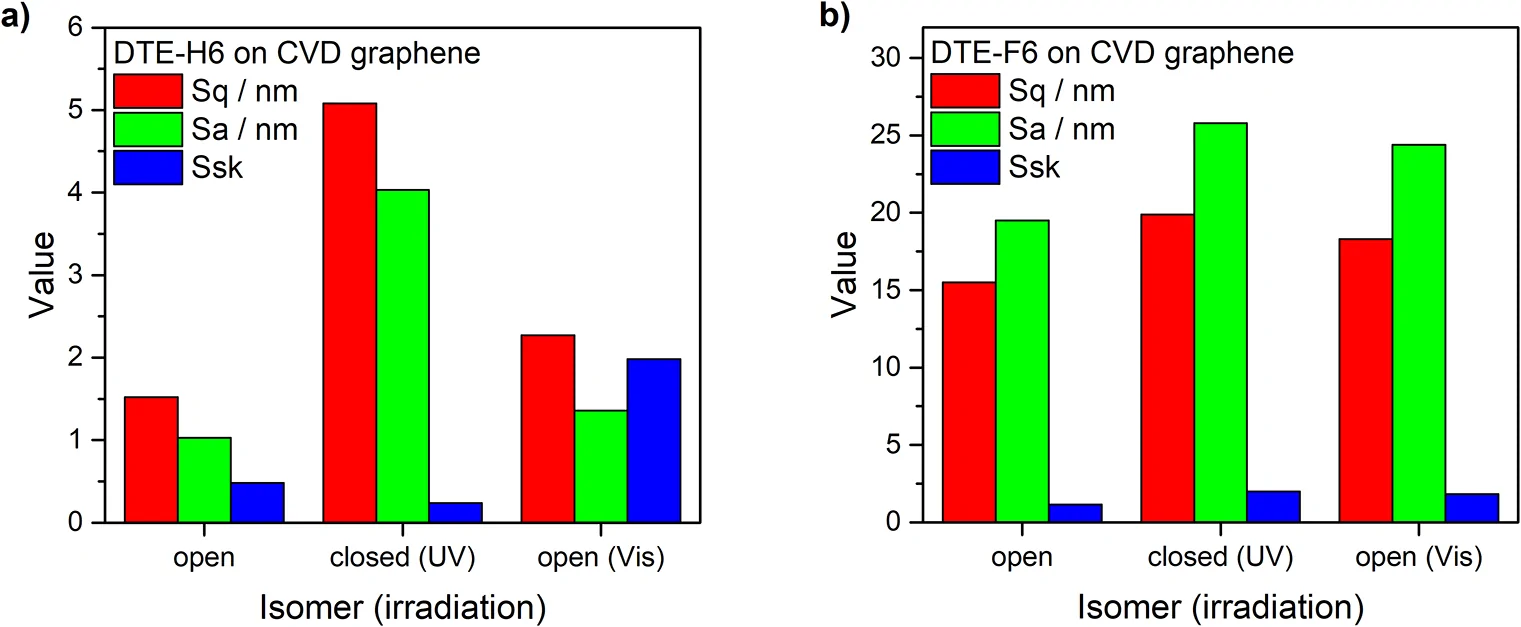
Statistical height parameters of (a) DTE-H6; (b) DTE-F6 thin films on CVD graphene, obtained by a statistical analysis of the AFM images, recorded upon irradiation.

Finally, the DTE/graphene transistors were assembled by transferring the CVD-grown graphene from copper to a SiO_2_/Si wafer via a polymer-assisted wet transfer process. Source and drain electrodes were patterned by photolithography and deposited by thermal evaporation, and the samples were subsequently treated under a vacuum to remove residual water. Thin films of both DTE molecules were spin-coated on top of the graphene devices, using the same experimental conditions as those used for the DTE/CVD graphene hybrids on a quartz substrate. The DTE-F6 device exhibits pronounced isomer-induced current modulation with good reversibility, whereas the DTE-H6 system shows a strong photoremanent response.

Figure 6 compares the transfer characteristics of the pristine graphene (black curve) with those of the DTE-F6-coated graphene with the photochromic layer being in the open (green) and closed (purple) states. The transfer characteristics of pristine graphene exhibit the characteristic V-shaped behavior of graphene field-effect transistors.^25^ Unfortunately, due to an unexpected premature local detachment of the DTE-F6 layer, we could only register two irradiation cycles. This is due to the excessive UV power and irradiation time, that have caused a similar problem to one already observed in the literature.^8^ The charge neutrality point (CNP) of the pristine graphene was (16.4 ± 0.4) V, which is the typical p-type doping, in graphene devices on SiO_2_ substrates, commonly attributed to charge traps and defect states at the graphene/SiO_2_ interface.^26^ After physisorption of the DTE-F6-o layer, the transfer exhibits a slight shift toward higher gate voltages, with a CNP of (17.6 ± 0.9) V, indicating that the open isomer slightly p-type dopes graphene. Upon UV irradiation, which induces the DTE-F6-o → DTE-F6-c photoisomerization, a clear shift of the CNP toward lower voltages is observed (12.8 ± 0.7) V, indicating the emergence of a noticeable n-type doping. Subsequent irradiation with visible (green) light restores the initial transfer characteristics. The recovery of the original CNP upon visible-light irradiation confirms a reversible and light-controlled modulation of the graphene doping.

**Figure 6:**
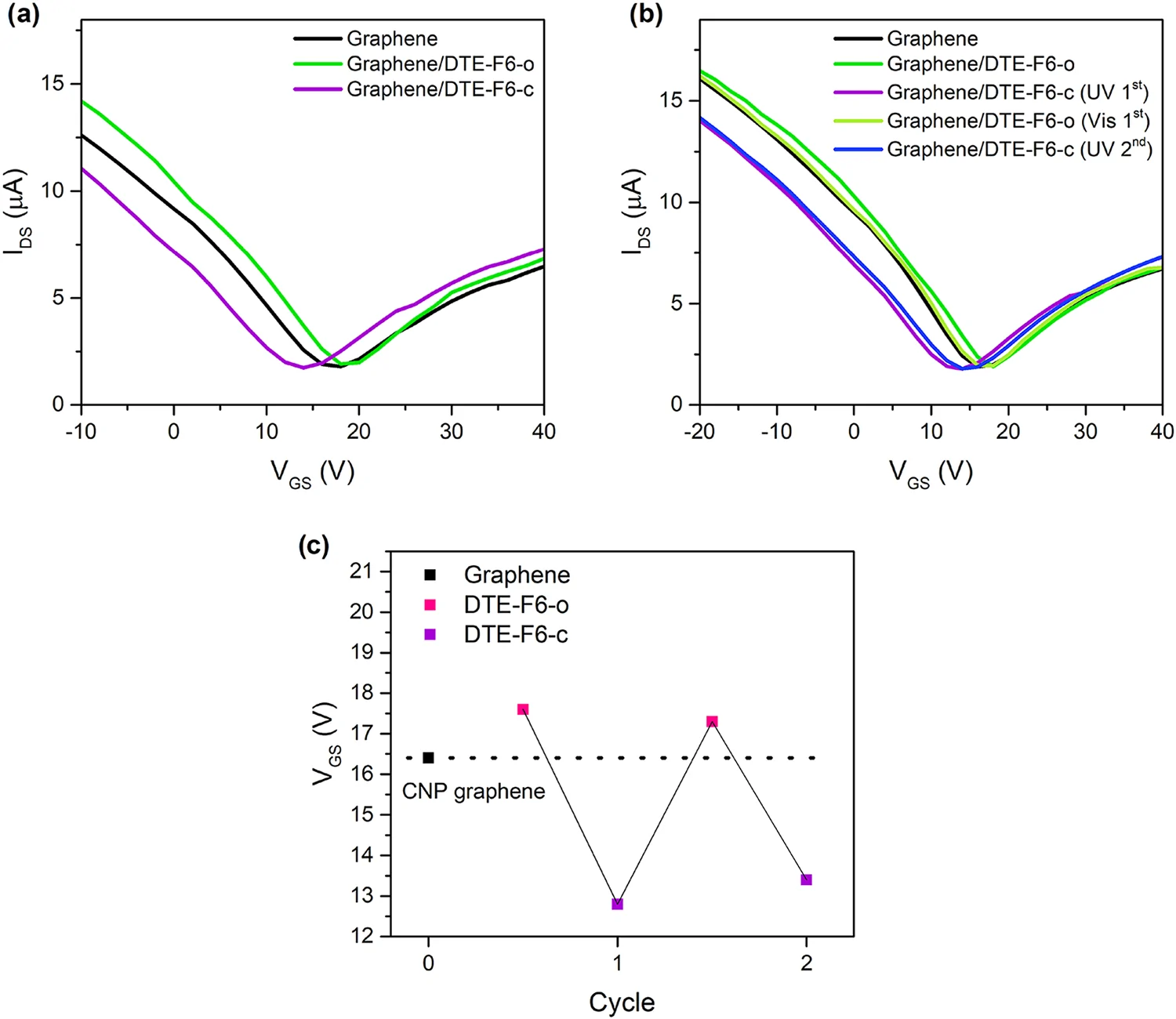
Transfer characteristics of DTE-F6/CVD graphene measured at *V*_DS_ = 0.01 V under a N_2_ atmosphere. Electrical characterization of the G-FET performance during: (a) one irradiation cycle; (b) few irradiation cycles; and (c) CNP values during few irradiation cycles.

## 4 Conclusions

In summary, we have studied the optical behavior of two dioctadecyl-substituted DTEs differing in the cyclopentene moiety functionalization by comparing a fully hydrogenated derivative with a fully fluorinated derivative. The perfluorinated derivative was found to outperform the hydrogenated one in its photoisomerization efficiency and cyclability in solution as well as in thin-film form on quartz and on CVD graphene. In addition, the DTE-F6 showed a smaller tendency to undergo morphological reorganization when subjected to photoisomerization. When integrated into FETs, the hybrid graphene/DTE-F6 films display a light-triggered, reversible doping effect. Such controlled photoresponsiveness opens the door to applications in light-sensing devices and neuromorphic computing.

## Supplementary Material



## Notes

Synthesis and NMR characterization of DTE-F6, UV–vis characterization of DTE-H6 and DTE-F6 solutions, absorbance spectra during isomerization of DTE-H6 and DTE-F6, and Raman spectra of DTE-F6-graphene FET (PDF)

## ⊥

Department of Engineering, University of Perugia, CIRIAF—Interuniversity Research Center, University of Perugia, Via G. Duranti 67, Perugia 06125, Italy

## #

Institut de Ciència de Materials de Barcelona (ICMAB-CSIC), Carrer dels Til.lers, 08193 Cerdanyola del Vallès, Spain.
