# Supplementary material for: Optically Responsive Field-Effect Transistors Based on Graphene/Diarylethene Hybrid Films
Source: ACS Omega. 2026 Aug 5;11(32):48700–6. doi: 10.1021/acsomega.6c07553 (PMC13491472; doi:10.1021/acsomega.6c07553)
Supplement: Supplementary file 1 [file ao6c07553_si_001.pdf]

Supporting information

Optically responsive field-effect transistors based on graphene/diarylethene hybrid films

Francesco Vinti,[#] Adrián Tamayo[§], Yeonsu Jeong, Piotr Cieciórski, Wojciech Danowski, Paolo Samorì*, Loredana Latterini*

Dr. F. Vinti, Prof. L. Latterini

Nano4Light Lab, Department of Chemistry, Biology and Biotechnology, University of Perugia, via Elce di Sotto 8, Perugia 06123, Italy

Email: loredana.latterini@unipg.it

Dr. F. Vinti, Dr. A. Tamayo, Prof. Y. Jeong, Prof. P. Samorì

University of Strasbourg & CNRS, ISIS & icFRC, 8 allee Gaspard Monge, 67000 Strasbourg, France.

Email: samori@unistra.fr

Prof. Dr. Y. Jeong

Institute of Quantum Science, Department of Physics, Inha University, 100 Inha-ro, Michuhol-gu, Incheon, 22212, Republic of Korea

P. Cieciórski, Prof. W. Danowski

Faculty of Chemistry, University of Warsaw, Ludwika Pasteura 1, 02-093 Warsaw, Poland

[#] Present address: Department of Engineering, University of Perugia, CIRIAF - Interuniversity Research Center, University of Perugia, Via G. Duranti 67, Perugia, 06125, Italy

[§] Present address: Institut de Ciència de Materials de Barcelona (ICMAB-CSIC), Carrer dels Til.lers, 08193 Cerdanyola del Vallès, Spain

Table of contents

1. Synthesis of DTE-F6-o and NMR data S-2

2. UV-Vis spectroscopy S-11

3. Raman spectroscopy S-13

4. References S-13

1. Synthesis of DTE-F6-o

All reagents were obtained from commercial sources (Sigma-Aldrich/Merck, TCI, AmBeed, BLDPharm) and used as received without further purification. Dry solvents were purchased from commercial suppliers (ThermoFisher Scientific – Acros). Column chromatography was performed on a Teledyne CombiFlash NextGen 300+ flash chromatography system. TLC: silica gel 60, Merck, 0.25 mm. High resolution mass spectrometry (ESI) was performed on a Shimadzu Q-TOF LCMS-9030 spectrometer with ESI ionization. NMR spectra were recorded on Bruker Avance III HD (^{1}H: 300 MHz, ^{13}C 100 MHz) or a Bruker Avance III HD (^{1}H: 500 MHz, ^{13}C 126 MHz). Chemical shifts are quoted in parts per million (ppm) relative to the residual solvent signal (for $CDCl_3$ δ 7.26 for ^{1}H, δ 77.16 for ^{13}C). For ^{1}H NMR spectroscopy, the splitting pattern of peaks is designated as follows: s (singlet), d (doublet), t (triplet), m (multiplet), br (broad), or dd (doublet of doublets). When specified, reactions were carried out under an inert gas atmosphere (argon, AirProducts, Argon Premier).

Perhydrodithienylethene[1] (**4**) and perfluorinated dithienylethene[2] (**5**) were synthesized following the previously established procedures.

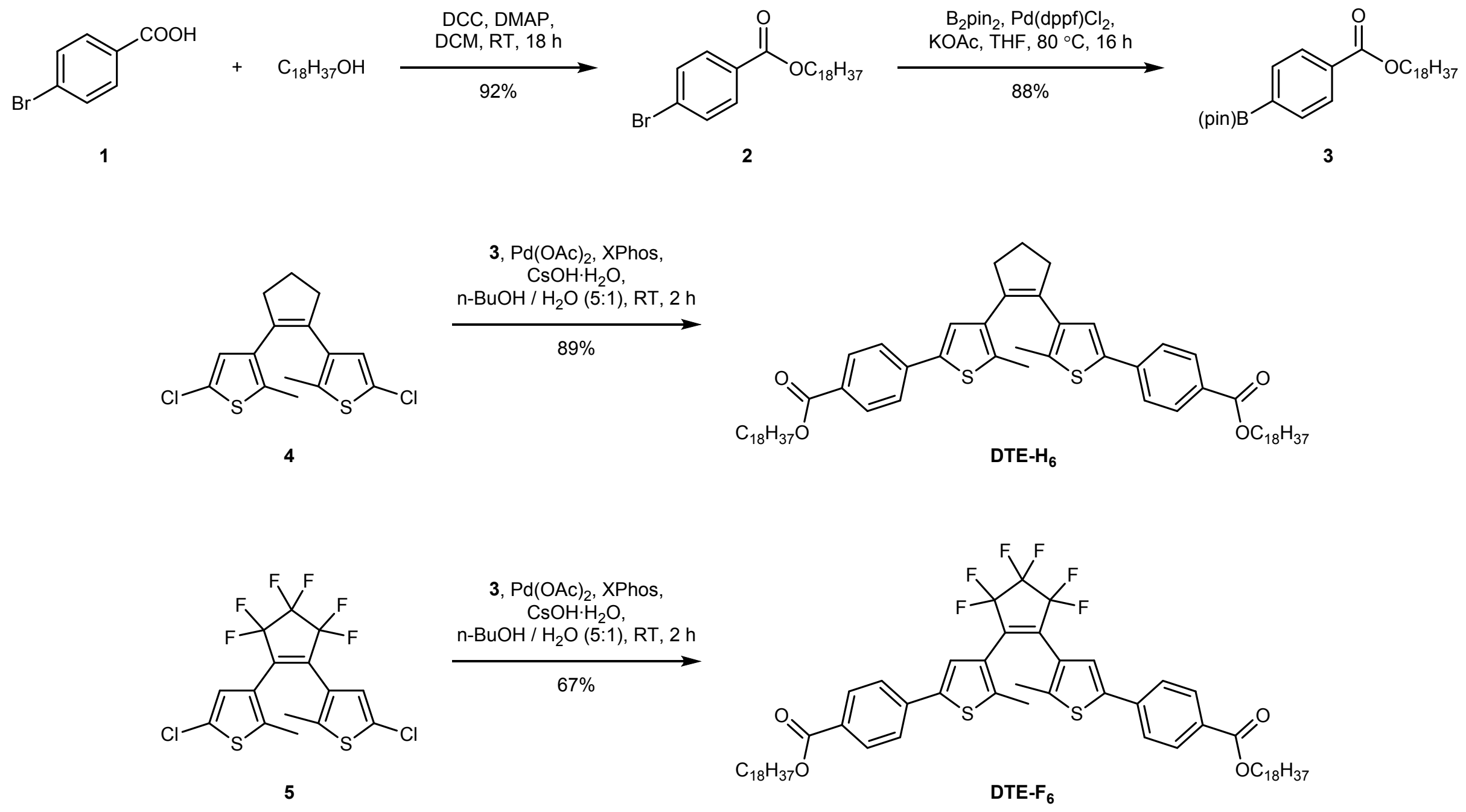

Scheme S1. Synthetic route followed for the synthesis of DTE-F6.

Octadecyl 4-bromobenzoate (2)

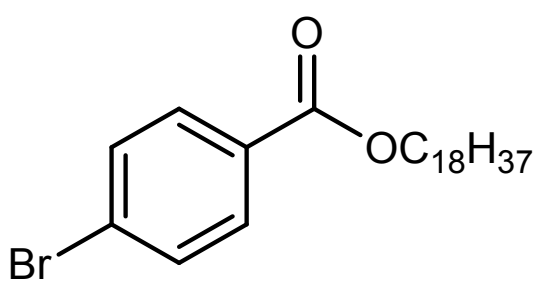

Octadecan-1-ol (10.0 g, 36.9 mmol, 1.0 equiv.), 4-bromobenzoic acid (**1**) (8.2 g, 40.7 mmol, 1.1 equiv.) and 4-(dimethylamino)pyridine (**DMAP**, 0,9 g, 7.4 mmol, 0.2 equiv.) were dissolved in 100 mL of DCM. A solution of N,N′-dicyclohexylcarbodiimide (**DCC**, 9.9 g, 48.1 mmol, 1.3 equiv.) in 40 mL of DCM was slowly added at room temperature via syringe, and the resulting mixture was allowed to stir at RT for 16 hours. Next, the reaction mixture was filtered through a celite pad, filtrate was concentrated in *vacuo*. The resulting solid was dissolved in a minimal amount of DCM and filtered through a short silica pad with DCM, and the filtrate was concentrated in *vacuo*. The crude product was crystallized from EtOH to afford octadecyl 4-bromobenzoate (**2**) (15.4 g, 34.0 mmol, 92%) as a white solid.

^{1}H NMR (500 MHz, $CDCl_3$) δ 7.93 – 7.87 (m, 2H), 7.61 – 7.55 (m, 2H), 4.30 (t, *J* = 6.7 Hz, 2H), 1.75 (dq, *J* = 8.3, 6.7 Hz, 2H), 1.46 – 1.38 (m, 2H), 1.26 (s, 28H), 0.88 (t, *J* = 7.0 Hz, 3H).

^{13}C NMR (126 MHz, $CDCl_3$) δ 166.1, 131.8, 131.2, 129.6, 128.0, 65.6, 32.1, 29.9, 29.8, 29.8, 29.8, 29.8, 29.7, 29.7, 29.5, 29.4, 28.8, 26.2, 22.8, 14.3. Not all aliphatic ^{13}C resonances of the C18 chain could be resolved

HRMS (ESI+): calcd for $C_{25}H_{42}BrO_2^+$ (M+H$^+$) 453.2363 found 453.2368

Octadecyl 4-(4,4,5,5-tetramethyl-1,3,2-dioxaborolan-2-yl)benzoate (**3**)

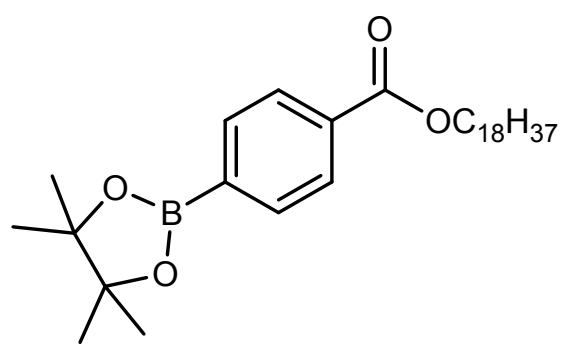

Under inert atmosphere, ester **2** (5.0 g, 11.0 mmol, 1.0 equiv.), [1,1′-bis(diphenylphosphino)ferrocene]dichloropalladium(II) (**Pd(dppf)Cl$_2$**, 0.4 g, 5 mol%, 0.55 mmol), bis(pinacolato)diboron (**B$_2$pin$_2$**, 3.35 g, 13.2mmol, 1.2 equiv.) and potassium acetate (**KOAc**, 3.25 g, 33.1 mmol, 3.0 equiv.) were suspended in 75 mL of anhydrous THF. The mixture was degassed by sparging with Ar for 5 mins. The reaction mixture was degassed by sparging with argon for 5 min, and the mixture was then heated to 80 °C and allowed to stir at this temperature for 16 h. Next, the mixture was cooled to RT, diluted with EtOAc and washed twice with H_2O, aqueous HCl (10% w/w), saturated aqueous $NaHCO_3$ and brine. The organic layer was dried over anhydrous $MgSO_4$, filtered, and concentrated in *vacuo*. The resulting solid was dissolved in DCM and filtered through a short -silica pad with DCM. The crude product was crystallized from MeOH to provide octadecyl 4-(4,4,5,5-tetramethyl-1,3,2-dioxaborolan-2-yl)benzoate (**3**) (4.8 g, 9.7 mmol, 87%) as a white solid.

^{1}H NMR (500 MHz, $CDCl_3$) δ 8.03 – 8.00 (m, 2H), 7.88 – 7.85 (m, 2H), 4.31 (t, *J* = 6.7 Hz, 2H), 1.82 – 1.72 (m, 2H), 1.46 – 1.39 (m, 2H), 1.31 (d, *J* = 51.2 Hz, 4oH), 0.91 – 0.84 (m, 3H).

^{13}C NMR (126 MHz, $CDCl_3$) δ 166.9, 134.8, 132.9, 128.7, 84.3, 65.4, 32.1, 29.9, 29.8, 29.8, 29.8, 29.7, 29.7, 29.5, 29.4, 28.8, 26.2, 25.0, 22.8, 14.3. Not all aliphatic ^{13}C resonances of the C18 chain could be resolved

HRMS (ESI+): calcd for $C_{25}H_{54}BO_4^+$ (M+H$^+$) 501.4121 found 501.4145

Dioctadecyl 4,4'-((perfluorocyclopent-1-ene-1,2-diyl)bis(5-methylthiophene-4,2-diyl))dibenzoate (DTE-F$_6$)

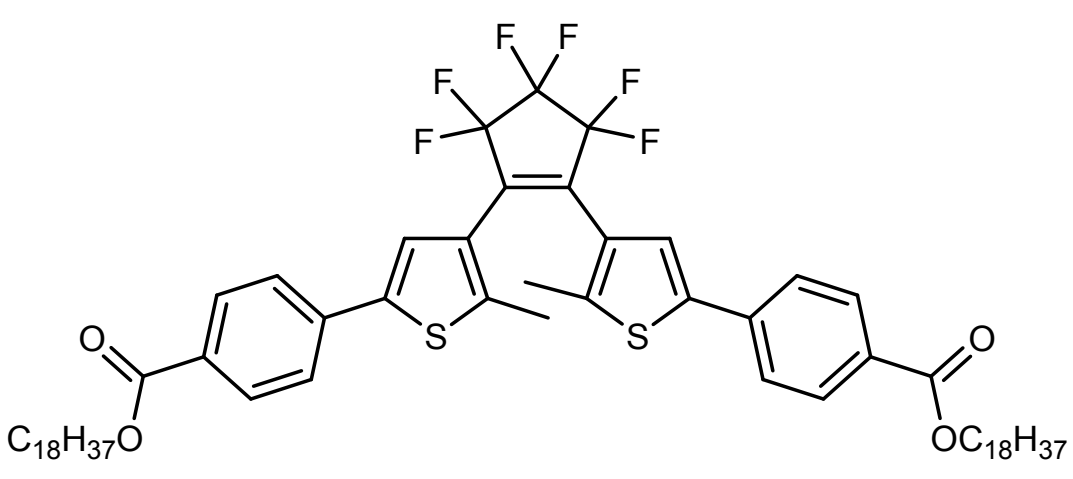

Under Ar atmosphere, **CsOH·H$_2$O** (0.39 g, 2.3 mmol, 3.4 equiv.) was dissolved in 1.2 mL of deionized water to give a 2 M solution, and the resulting solution was degassed by sparging with Ar for 20 min. In a separate Schlenk flask, under Ar atmosphere, dithienylethene (**5**) (0.5 g, 1.5 mmol, 1.0 equiv.), boronic acid pinacol ester (**3**) (1.67 g, 3.3 mmol, 2.2 equiv), palladium(II) acetate (**Pd(OAc)$_2$**, 15.4 mg, 68.6 μmol, 6 mol%) and 2-dicyclohexylphosphino-

2′,4′,6′-triisopropylbiphenyl (**XPhos**, 38.2 mg, 80.1 µmol, 7 mol%) were suspended in 6 mL of degassed *n*-BuOH. The mixture was gently heated with a heat gun until all reagents dissolved, then allowed to stir without further heating for 10 min. Next, the 1.2 mL of pre-prepared CsOH solution was added to this mixture via syringe and the reaction mixture was allowed to stir at RT for 2 h. The reaction mixture was diluted with DCM and extensively washed with H_2O; brine dried over $MgSO_4$, filtered, and concentrated in vacuo. The crude product was purified by flash column chromatography (SiO_2, petroleum ether/Et_2O) to afford **DTE-F6** (0.51 g, 0.46 mmol, 67%) as a white solid.

^{1}H NMR (500 MHz, $CDCl_3$) δ 8.11 – 7.99 (m, 4H), 7.65 – 7.53 (m, 4H), 7.38 (s, 2H), 4.33 (t, *J* = 6.7 Hz, 4H), 2.00 (s, 6H), 1.77 (dt, *J* = 14.7, 6.8 Hz, 4H), 1.49 – 1.40 (m, 4H), 1.26 (s, 56H), 0.91 – 0.86 (t, *J* = 6.9 Hz, 6H).

^{19}F NMR (471 MHz, $CDCl_3$) δ -110.03 (t, *J* = 5.2 Hz), -131.82 (p, *J* = 5.2 Hz). ^{19}F NMR spectrum is unreferenced.

^{13}C NMR (126 MHz, $CDCl_3$) δ 166.3, 142.8, 141.3, 137.4, 130.5, 129.8, 126.2, 125.4, 123.9, 65.4, 32.1, 29.9, 29.8, 29.8, 29.8, 29.7, 29.7, 29.5, 29.4, 28.9, 26.2, 22.8, 14.8, 14.3. Not all aliphatic ^{13}C resonances of the C18 chain could be resolved. ^{13}C resonances associated with the perfluorinated cyclopentene moiety could not be observed due to the coupling with ^{19}F nuclei.

HRMS (ESI+): calcd for $C_{65}H_{90}F_6O_4S_2Na^+$ (M+Na$^+$) 1135.6077 found 1135.6069

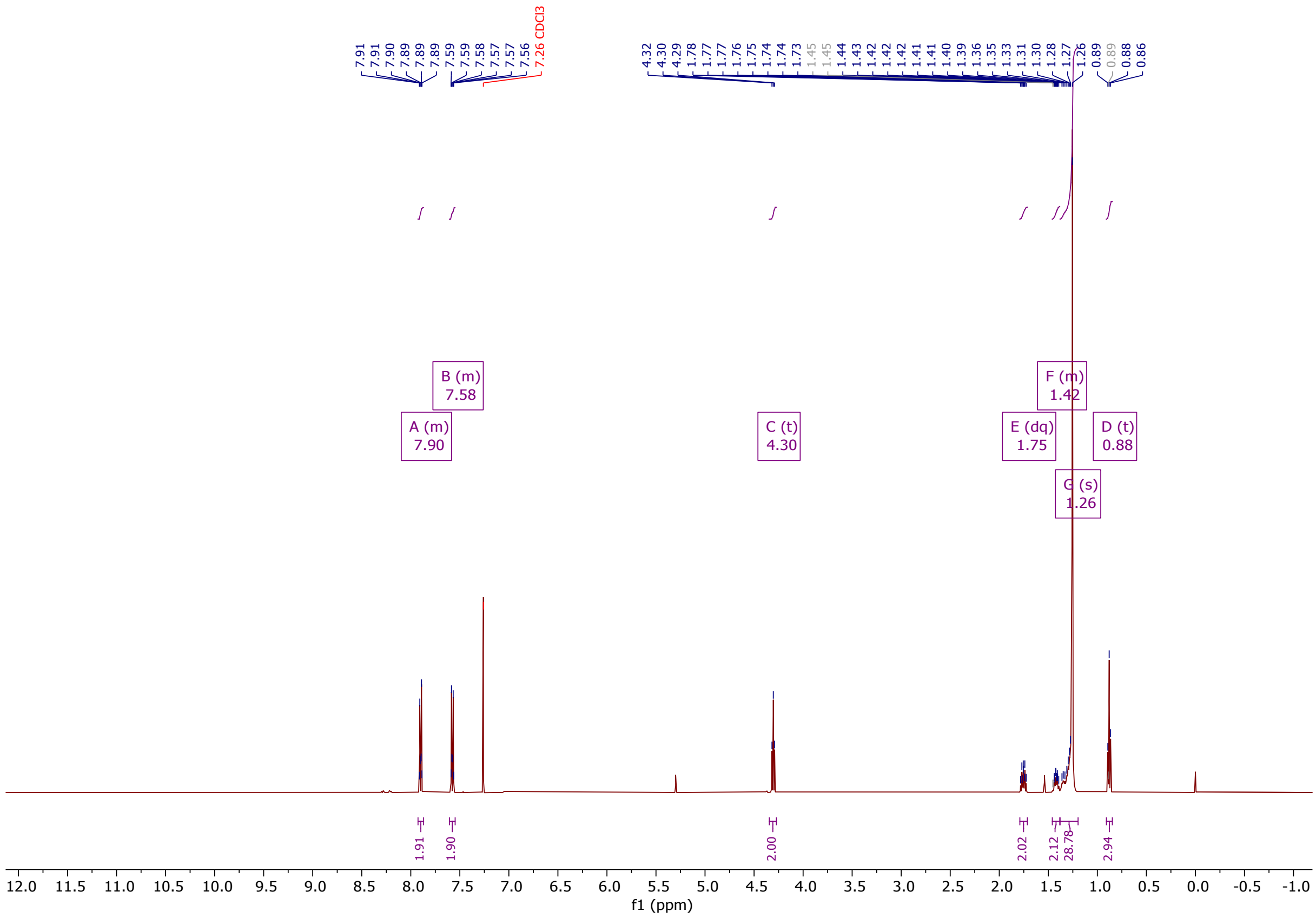

Figure S1. ^{1}H NMR (500 MHz, $CDCl_3$) spectra of **2**.

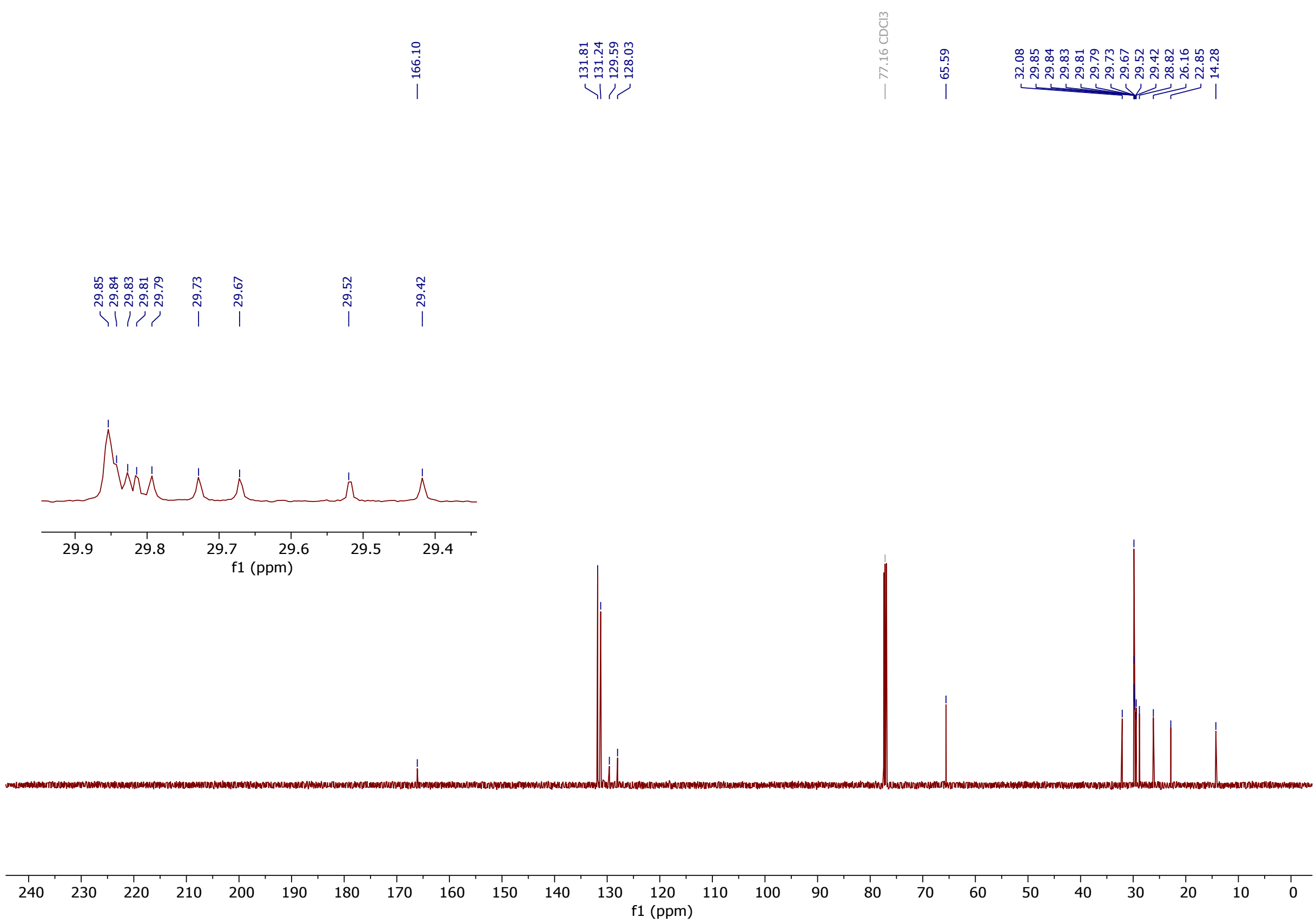

Figure S2. ^{13}C NMR (126 MHz, $CDCl_3$) spectrum of **2**.

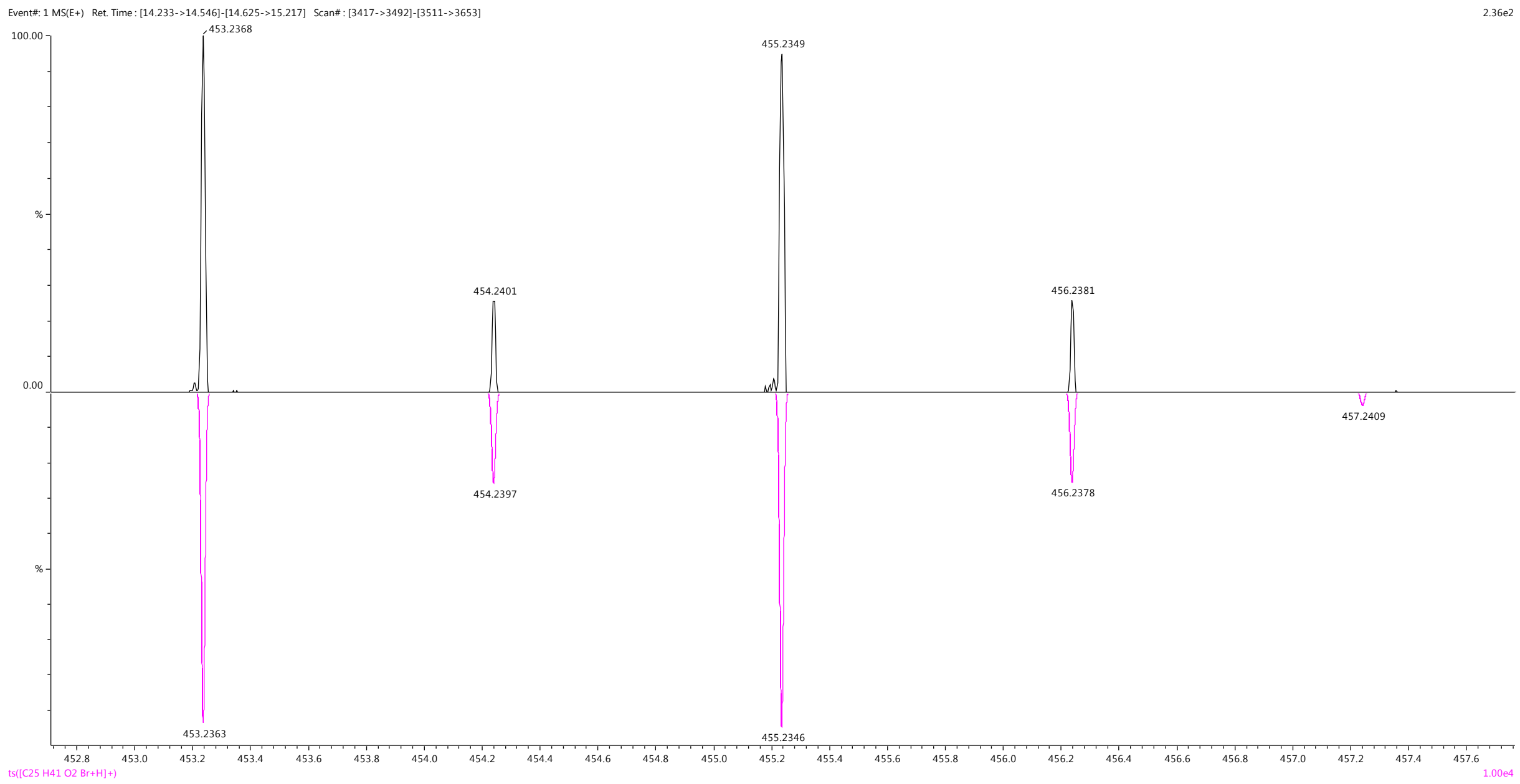

Figure S3. HRMS (ESI+) spectrum of **2** (top: measured, bottom: cacld).

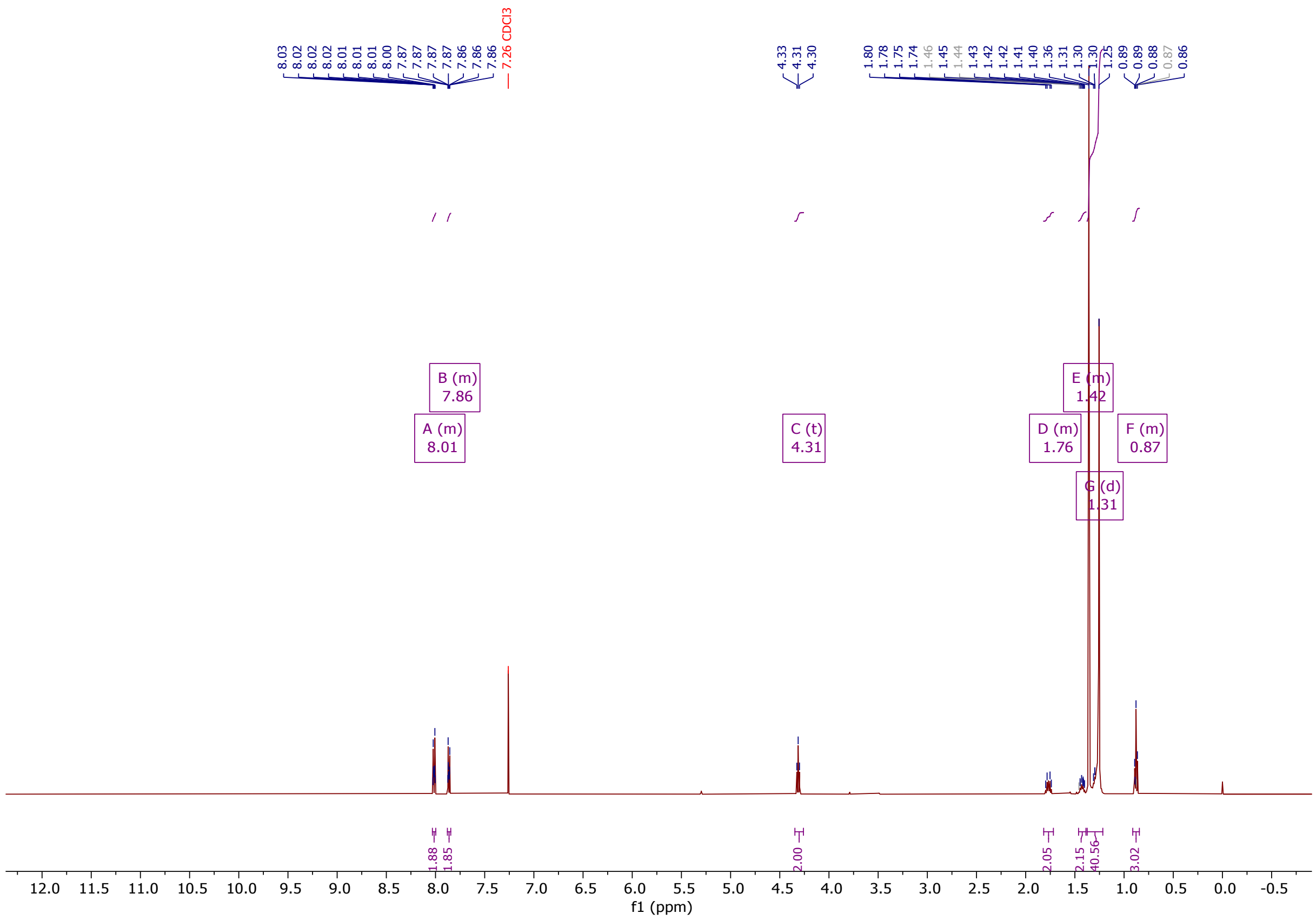

Figure S4. ^{1}H NMR (500 MHz, $CDCl_3$) spectrum of **3**.

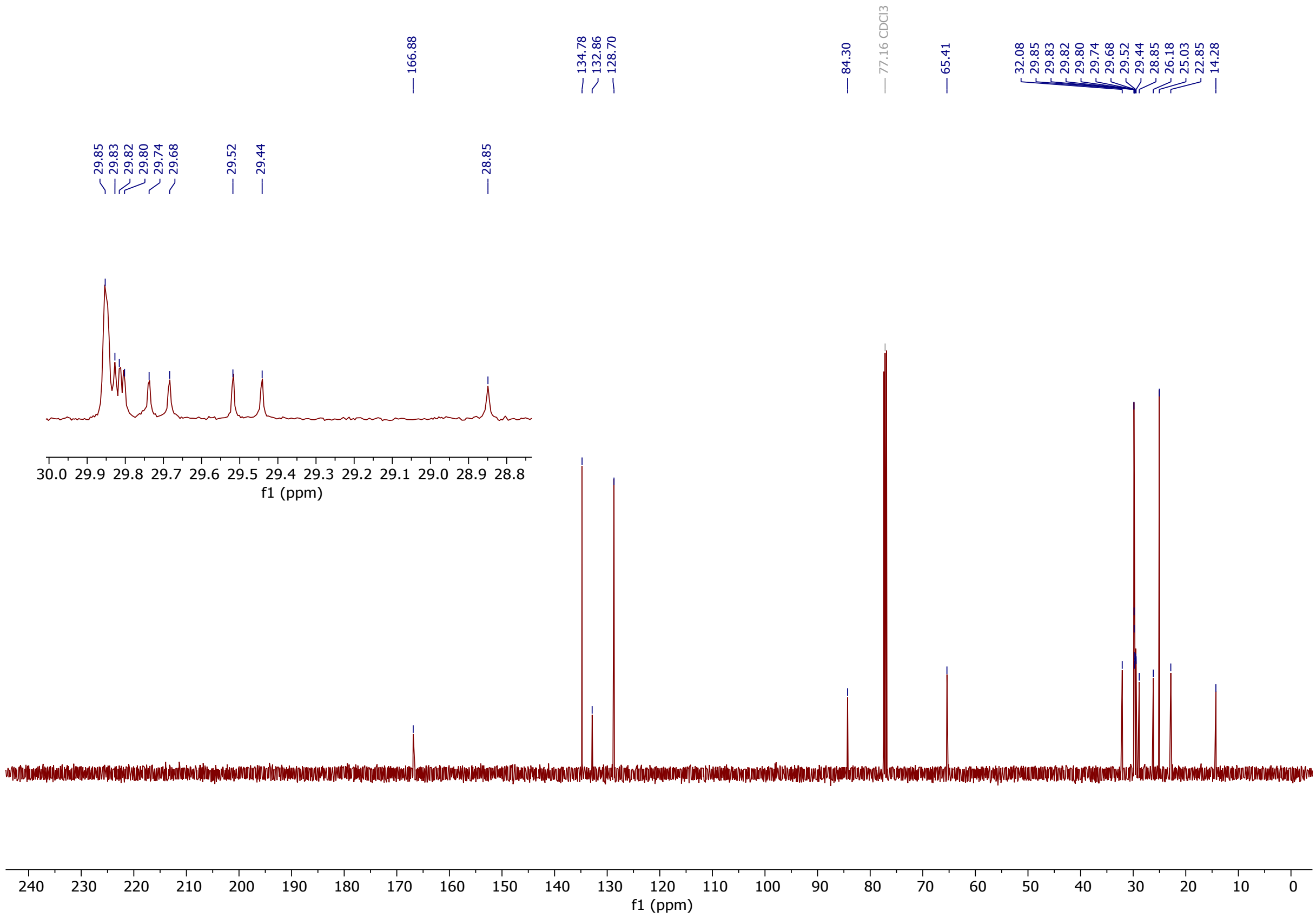

Figure S5. ^{13}C NMR (126 MHz, $CDCl_3$) spectrum of **3**.

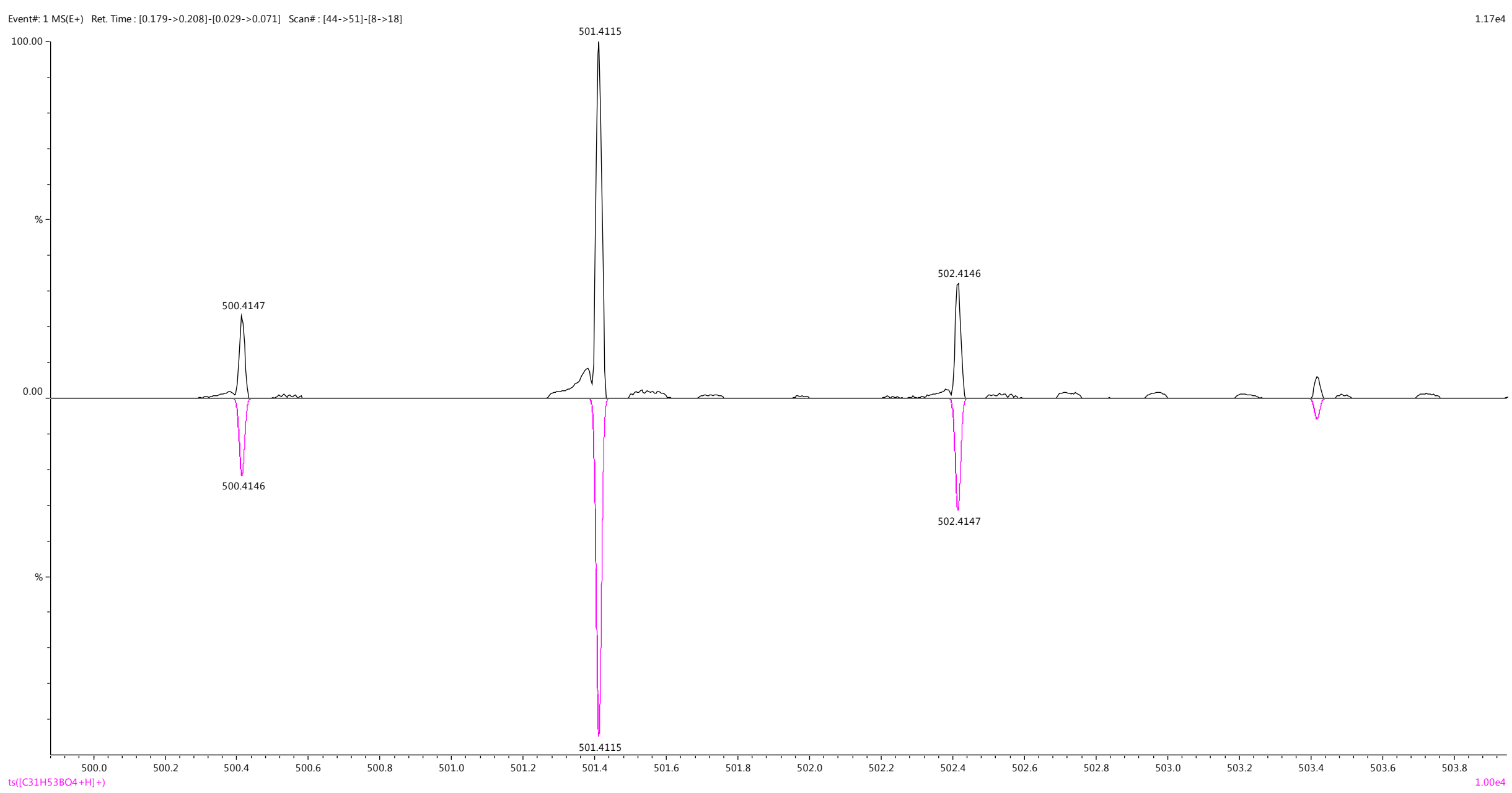

Figure S6. HRMS (ESI+) spectrum of **3** (top: measured, bottom: cacld).

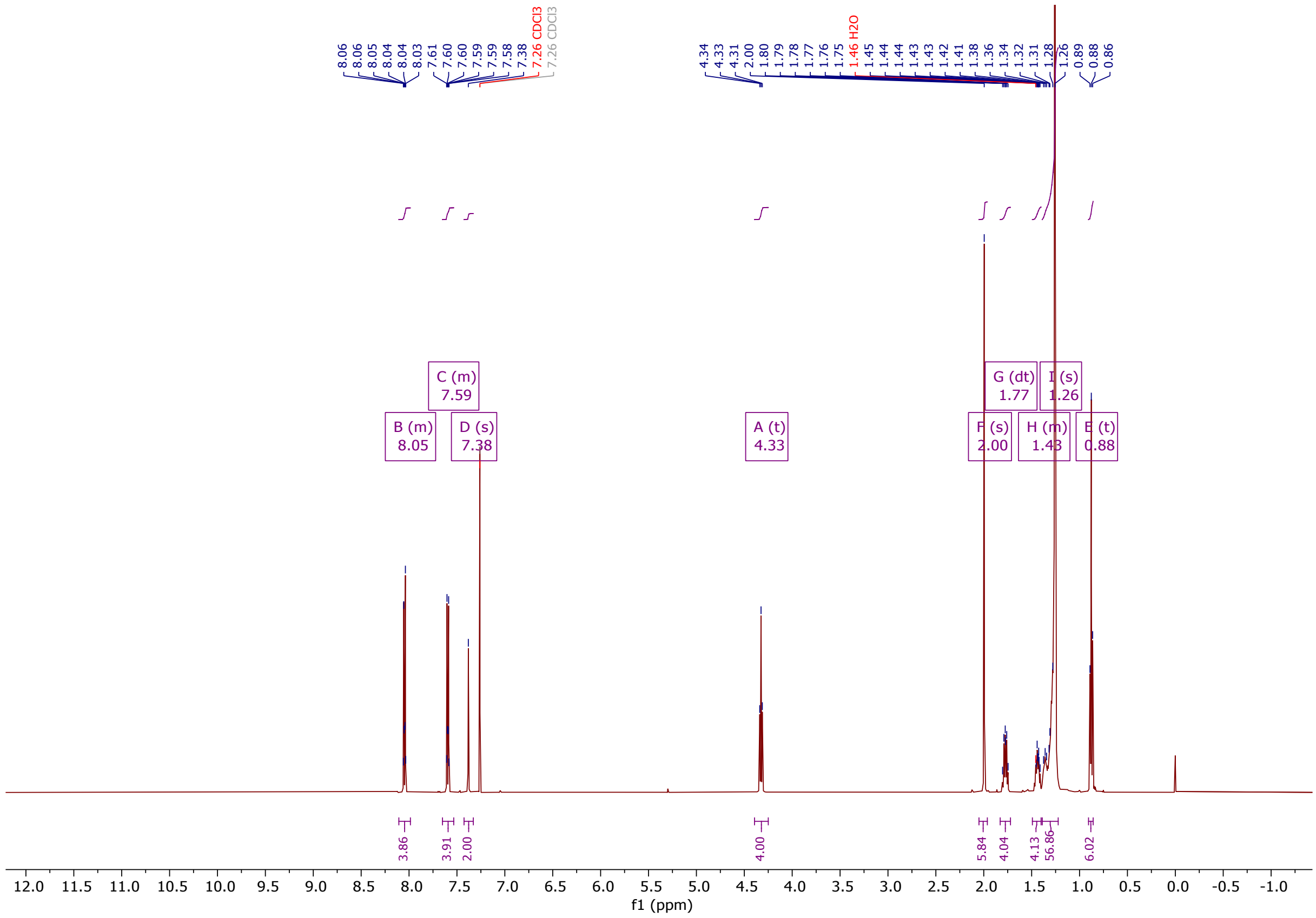

Figure S7. ^{1}H NMR (500 MHz, $CDCl_3$) spectrum of **DTE-F$_6$**.

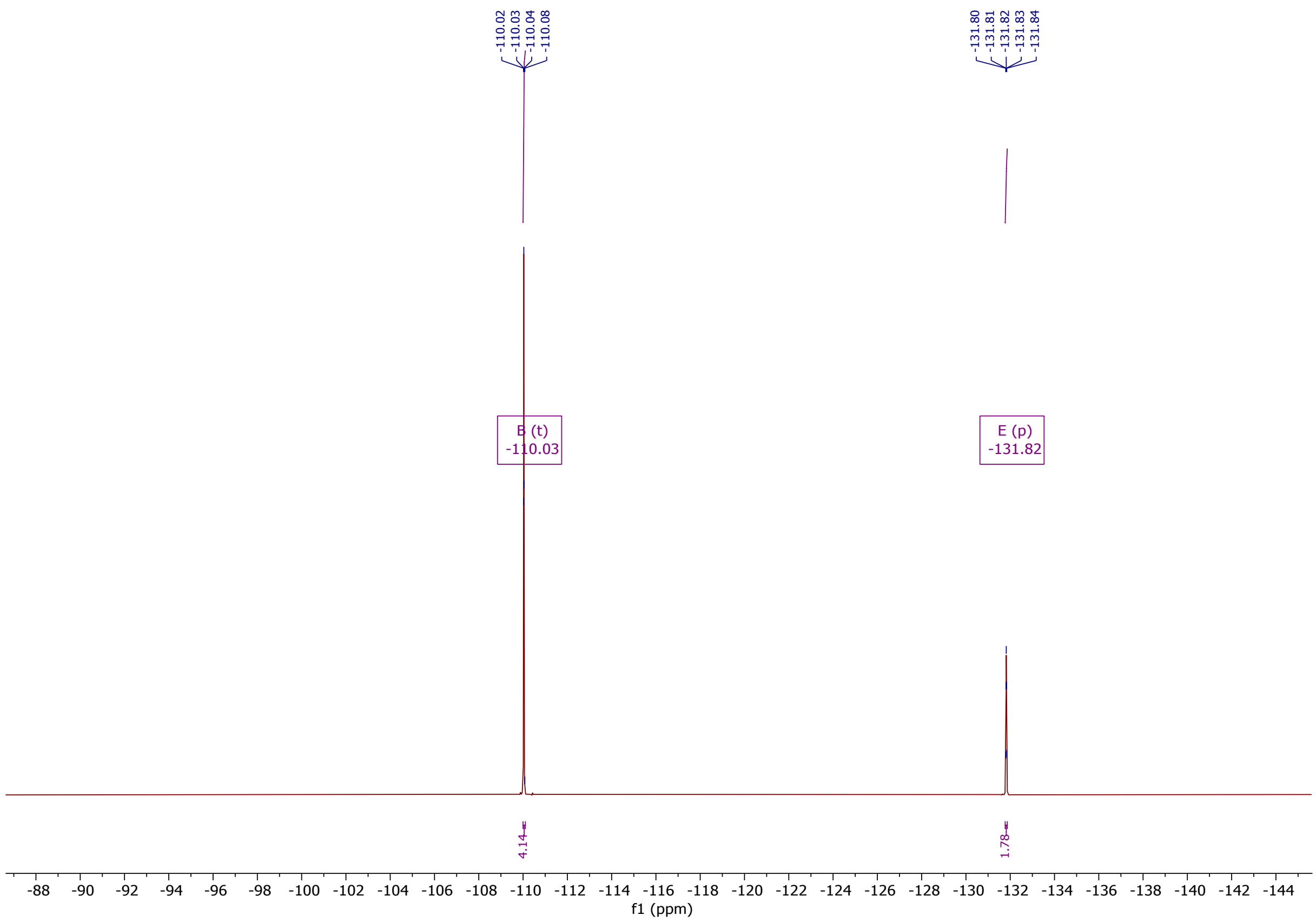

Figure S8. ^{19}F NMR (471 MHz, $CDCl_3$) spectrum of **DTE-F$_6$**.

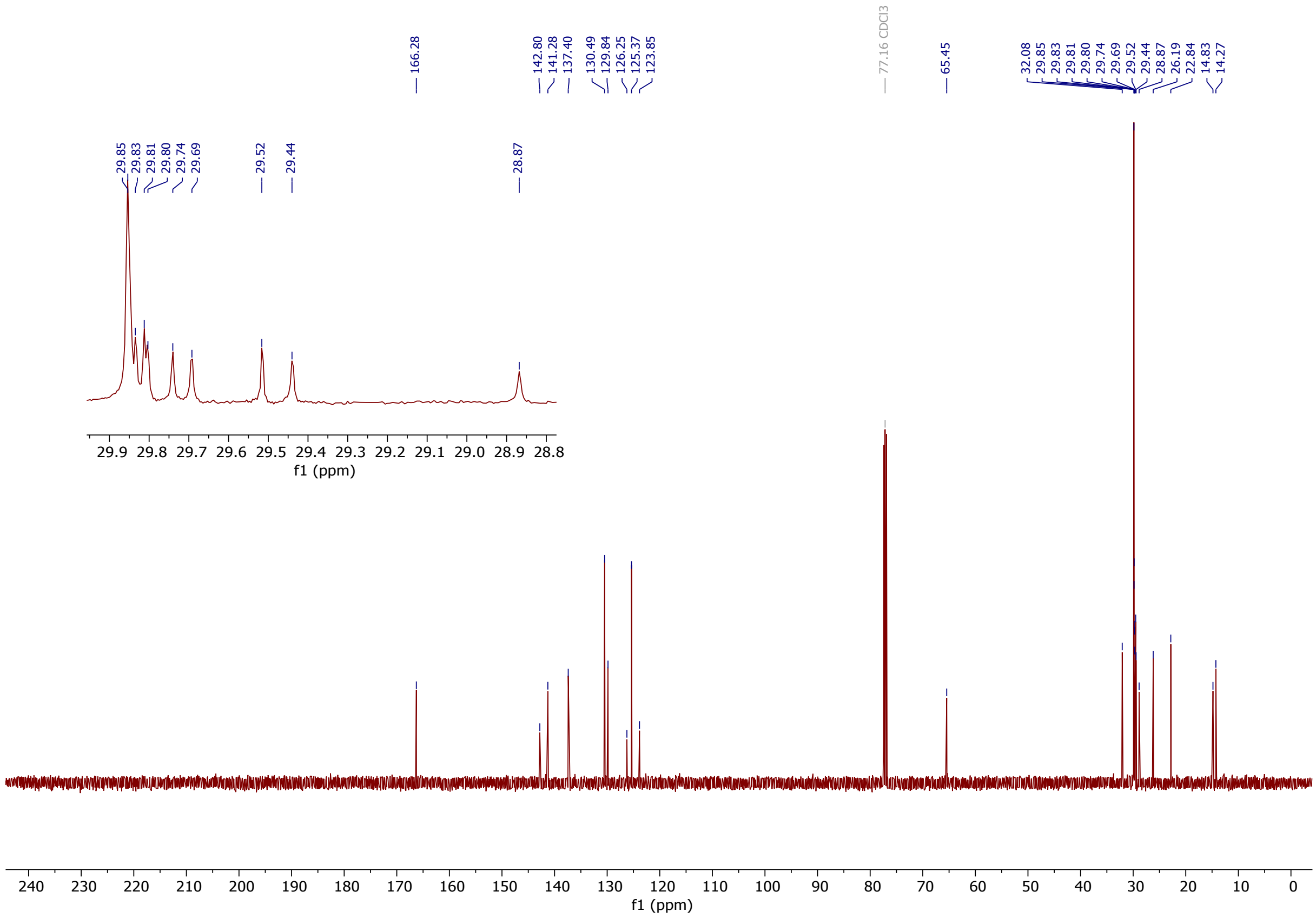

Figure S9. ^{13}C NMR (126 MHz, $CDCl_3$) spectrum of **DTE-F$_6$**.

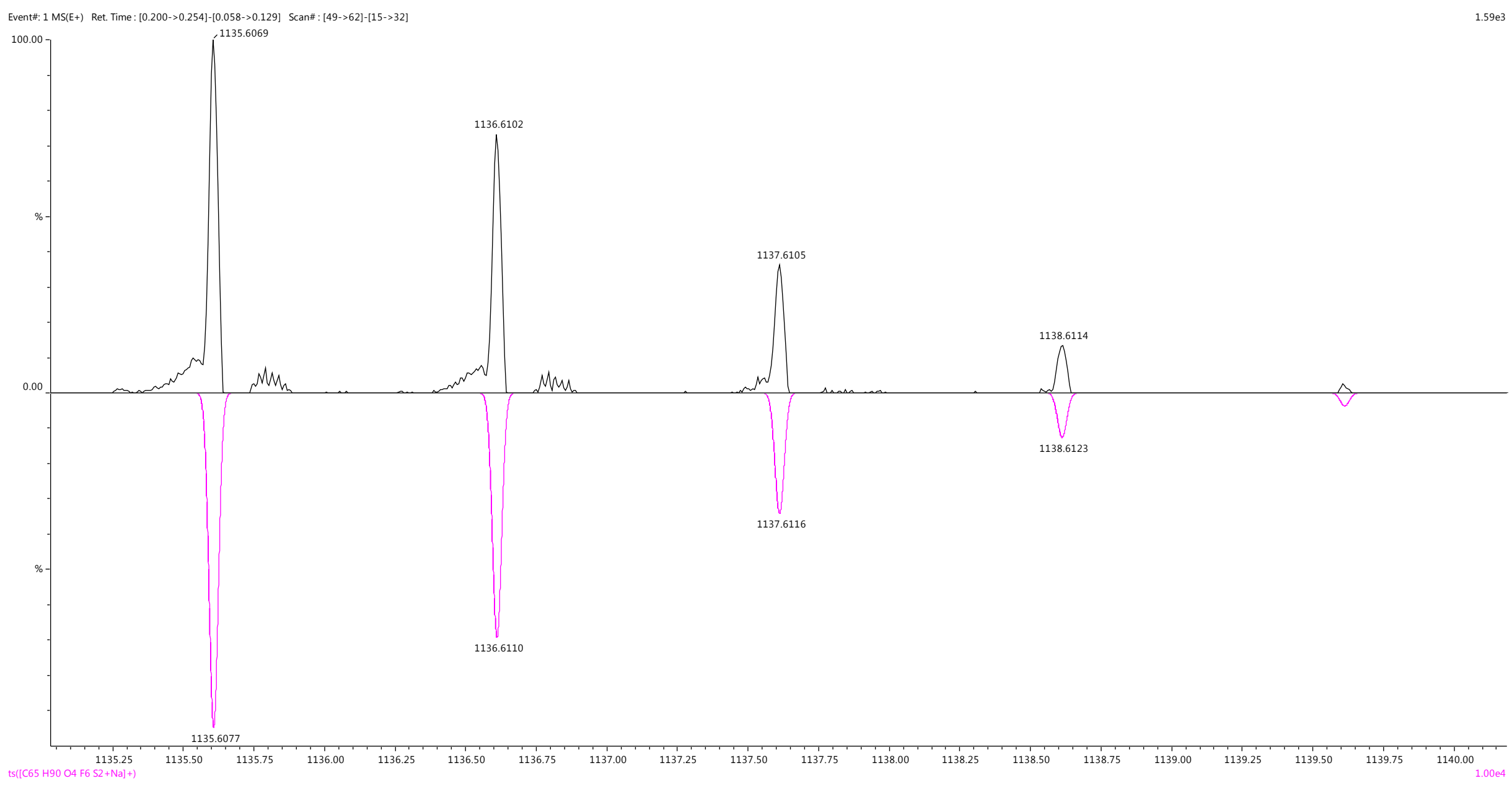

Figure S10. HRMS (ESI+) spectrum of **DTE-F$_6$** (top: measured, bottom: cacld).

2. UV-Vis spectroscopy

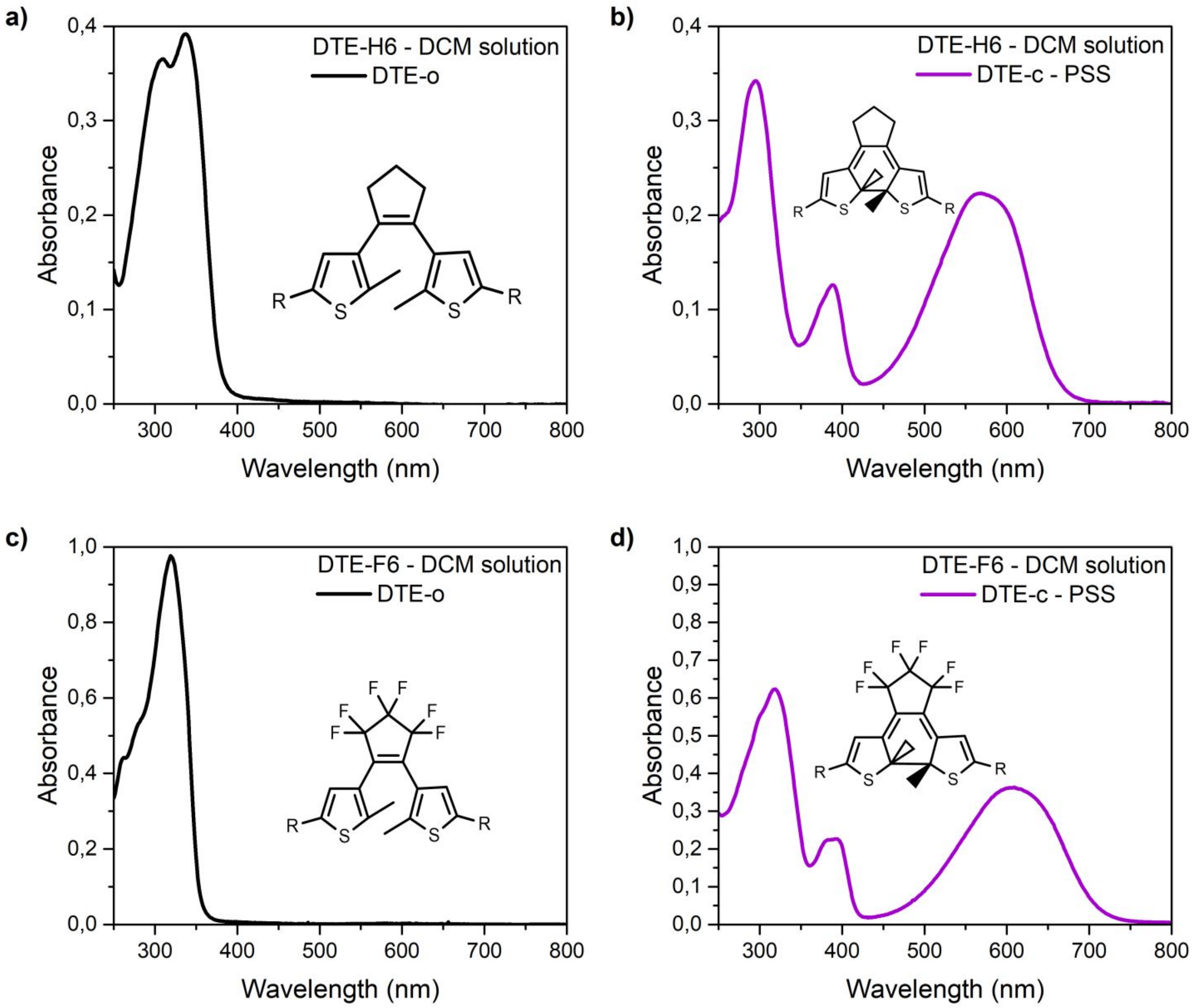

Figure S11. Absorbance spectra of DCM solution of: **a)** DTE-H6 open isomer; **b)** DTE-H6 closed isomer (PSS); **c)** DTE-F6 open isomer; **d)** DTE-F6 closed isomer (PSS).

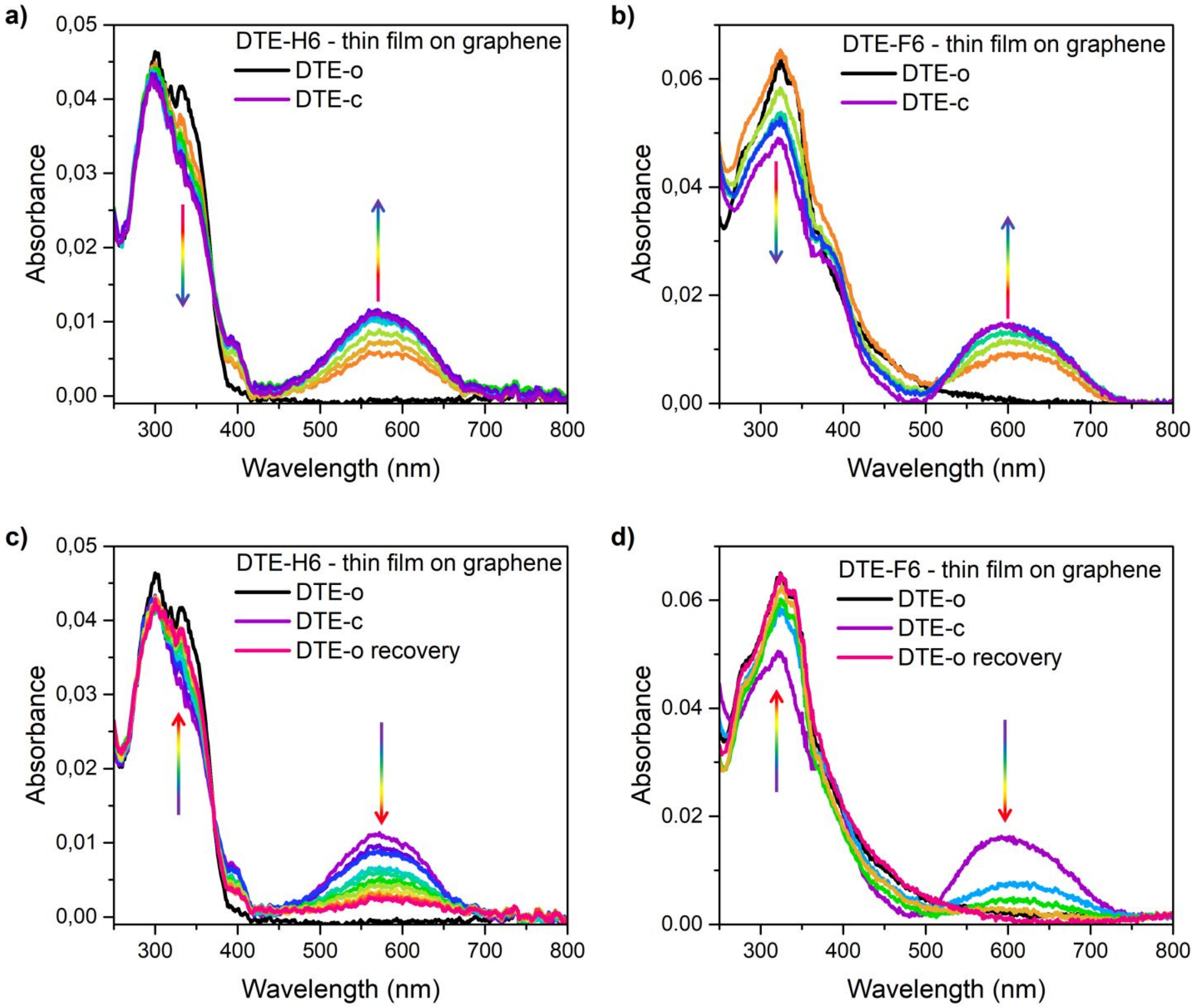

Figure S12. Absorbance spectra obtained during successive irradiation, of the data reported in **Figure 2**; a and c) DTE-H6; b and d) DTE-F6.

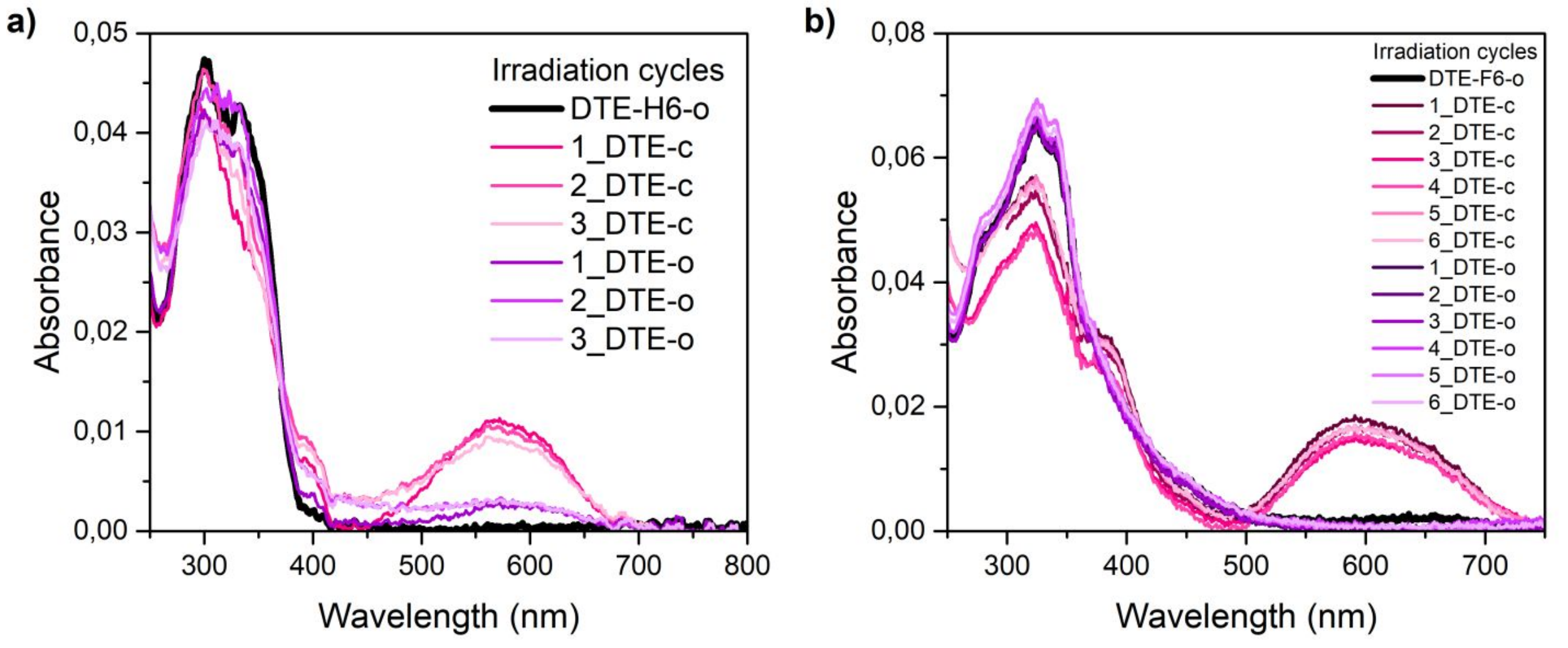

Figure S13. Complete absorbance spectra of the open and closed isomer of the data reported in **Figure 3**; a) DTE-H6; b) DTE-F6.

3. Raman spectroscopy

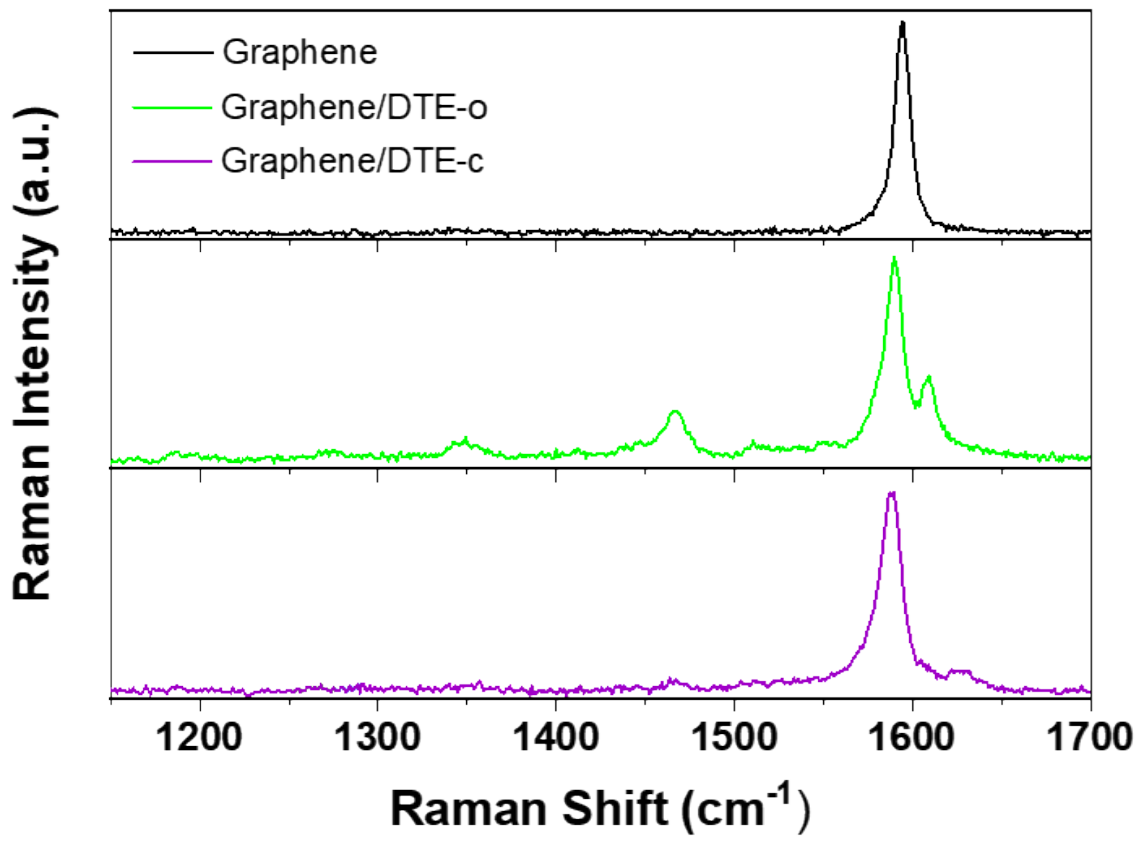

Figure S14. Raman spectra of graphene, graphene/DTE-F6-o and graphene/DTE-F6-c. Pristine graphene exhibits the characteristic G band at 1580 cm^{-1}.

4. References

1. Bonacchi, S. *et al.* Surface-Induced Selection During In Situ Photoswitching at the Solid/Liquid Interface. *Angewandte Chemie* **127**, 4947–4951 (2015).

2. Hermes, S. *et al.* New fast synthesis route for symmetric and asymmetric phenyl-substituted photochromic dithienylethenes bearing functional groups such as alcohols, carboxylic acids, or amines. *Tetrahedron Lett.* **50**, 1614–1617 (2009).
